# An experimental approach to the preservation potential of magnetic signatures in anthropogenic fires

**DOI:** 10.1371/journal.pone.0221592

**Published:** 2019-08-29

**Authors:** Ángela Herrejón Lagunilla, Ángel Carrancho, Juan José Villalaín, Carolina Mallol, Cristo Manuel Hernández

**Affiliations:** 1 Departamento de Física, Universidad de Burgos, Burgos, Spain; 2 Área de Prehistoria, Departamento de Historia, Geografía y Comunicación, Universidad de Burgos, Burgos, Spain; 3 Departamento de Geografía e Historia, Área de Prehistoria (Facultad de Humanidades), Universidad de La Laguna, Campus de Guajara, La Laguna, Tenerife, Spain; 4 Archaeological Micromorphology and Biomarkers (AMBI Lab), Instituto Universitario de Bio-Orgánica Antonio González, La Laguna, Tenerife, Spain; Universita degli Studi di Milano, ITALY

## Abstract

Archaeomagnetic and rock-magnetic methods are of great value in the identification of archaeological fire, especially in Palaeolithic sites where evidence is usually scarce, ambiguous or poorly preserved. Although taphonomic processes can significantly modify Palaeolithic combustion structures, the extent to which such processes affect the magnetic record remains unknown. Here we report the results of an archaeomagnetic study involving five, two-to-five-year-old experimental combustion structures in open-air and cave settings. Some of these combustion structures involved post-combustion human actions such as trampling and relighting. Our results show pseudo-single domain (PSD) magnetite as the main magnetic carrier. Wood ash layers of combustion structures are the most magnetic facies followed by thermally altered sediments constituting the combustion substrates. A decreasing magnetic concentration pattern in depth was observed as a function of temperature. Positive correlation was found between good-quality directional data and macroscopically well-preserved combustion structures. Partial thermoremanent magnetization (pTRM) was the main magnetization mechanism identified in the combustion substrate facies. These data coupled with partial thermomagnetic curve experiments show the potential of these methods to estimate maximum temperatures of the last combustion event. Relightings show very good directional results, but they cannot be identified because the time between them is not enough to statistically distinguish directional variations of the local Earth´s magnetic field. The substrate sediment of an intensively trampled combustion structure yielded reliable archaeomagnetic directions. The results are discussed in terms of magnetization preservation potential and the effects of taphonomic processes on the archaeomagnetic record.

## 1. Introduction

Palaeolithic combustion features may generally comprise charcoal concentrations, calcitic wood ash or thermally altered sediment. Sometimes these constituents are found in stratigraphic association in what is referred to as a combustion structure [1]. In the last years, the characterization of Palaeolithic fire has been at the core of many multidisciplinary geoarchaeological studies (e.g.: [1–25]). Fire played a key role in human evolution (e.g.: [4,23,26–29]) and in order to approach the first stages of its use and control it is important to understand Palaeolithic combustion feature formation processes. In addition, archaeological fire and its products, including thermally altered sediment, can provide important clues about Palaeolithic behaviour and information about the integrity of the archaeological record (e.g.: [25,30–33]). This information is of great interest for the dissection of Palaeolithic palimpsests associated with combustion structures. Palimpsests are usually sedimentary deposits, variably rich in archaeological remains, representing an unknown number of human occupations episodes. Although these are normally excavated as single assemblages, they may correspond to successive anthropogenic accumulations [34]. It is possible to infer time and isolate human occupation episodes through a multidisciplinary approach to archaeological combustion structures (e.g.: [32,35,36]). Nevertheless, ethnoarchaeological, experimental and archaeological data that can be used as reference is still scarce [1].

Experimental approaches to the study of prehistoric fire have been fruitful (e.g.: [2,11,24,32,37–53]). They have provided valuable information on combustion structure formation aspects with particular emphasis on observable features such as temperature ranges, size and morphology, heat penetration and internal stratigraphy in relation to variables such as fuel type, combustion duration or different post-combustion actions (trampling, relighting…etc.). Despite these advances, certain issues remain unresolved. These include: determination of temperatures attained in simple hearths (e.g.: [51,53,54]), identification of relighting events [44] and the effect of taphonomic processes on the archaeological fire record [30,42,43,55].

Burning temperatures have been approached experimentally through different proxies including: chromatic variations and breakage of lithic artifacts (e.g.: [56]), colour and structural changes in bone (e.g.: [57–61]), carbon stable isotope composition [62] and magnetic parameters (e.g.: [54,63–65]), as well as sedimentary signatures detected through Fourier transformed infrared spectroscopy (e.g.: [66]) or thermoluminiscence (e.g.: [54,67,68]). However, these proxies are only applicable over a limited temperature range and their level of precision is variable. Also, thermal impact on different zones of a combustion structure (center vs. periphery or surface vs. subsurface) and on different constituents (ash, substrate, bones, etc.) varies considerably.

Regarding identification of relighting events in Palaeolithic combustion structures, repeated use of combustion structures has been suggested for some archaeological sites (e.g. [10,69]) and experimental studies have replicated reheated structures [44,50,70]. However, identification of different burning events in a single combustion structure has not been accomplished. One exception is a case in which relighting of an experimental combustion structure was identified through soil micromorphology. However, this was only possible thanks to the presence of a visible deposit (a bed of leaves) between the relightings [44].

Finally, another issue that remains unresolved is the effect of taphonomic processes on the archaeological fire record. The majority of experimental combustion structures are usually sampled and analyzed shortly after the burning event, not allowing enough time for taphonomic processes to take action on intact or anthropogenically modified (e.g., trampled [42,43],) fires. Certainly, it is not possible to replicate an archaeological time scale, but perhaps experiments lasting several years could address this problem, given that atmospheric and biogenic agents act on fresh deposits within months.

Archaeomagnetism and rock magnetism may help address the unresolved issues mentioned above. Experimental studies based on archaeomagnetism and rock magnetism have been carried out to test the applicability of these methods to identify burnt materials (e.g.: [71]), to characterize the magnetic properties of different facies of within combustion structures (e.g.: [72–74]), to infer the magnetization mechanisms (e.g.: [75–77]) and to assess the reliability of the Earth’s magnetic field record in burnt materials (e.g.: [75,78]). These works support magnetism-based methods as reliably applicable tools to solve questions of archaeological interest. Previous applications of the methods include paleotemperature determinations (e.g.: [54,55,63–65,79]), as well as identification of burning episodes (e.g. [16,71,80]), detection mechanical post-depositional processes affecting combustion structures (e.g.: [30,55,79]) and distinction of occupation events in Palaeolithic hearth-related assemblages [35]. However, the issues of relighting event identification and the effect of taphonomic processes on the fire record have not been explored so far.

Assuming that the direction of the Earth´s magnetic field changes with time, experimental relighting events carried out a few years apart can be evaluated through archaeomagnetic analysis. This has never been attempted. Nor has there been any study to understand how the magnetic signal (directional record and magnetic properties) is preserved through time in combustion structures affected by different taphonomic processes.

To the best of our knowledge, there are no actualistic experimental studies focusing on the effects of taphonomic processes on the magnetic directional record of combustion structures and their variation in magnetic properties. Assessing the effects of the taphonomic processes in different facies and substrates would allow us to evaluate which are the most suitable materials for the application of archaeomagnetic analyses on similar contexts (i.e. Palaeolithic sites). This is relevant because archaeomagnetic studies on combustion structures can shed light on problems involved in Palaeolithic palimpsest dissection [35]. Moreover, the detection of possible specific patterns in the magnetic behaviour related to different taphonomic processes might help us identify such processes in the archaeological record.

Here we report a systematic archaeomagnetic study of five experimental combustion structures that were left untouched for two to five years before their sampling and analysis. These combustion structures were made as part of the Neanderthal Fire Technology Project (Leakey Foundation) for the investigation of different aspects of Paleolithic fire and its material manifestations. Our main goal here is to assess the reliability of the Earth’s magnetic field (EMF) direction as recorded by natural remanent magnetization (NRM) under different taphonomic conditions and further implications in terms of archaeological fire identification, with emphasis on thermal characterization of combustion structures and relighting event assessment.

## 2. Materials and methods

**Fig 1:**
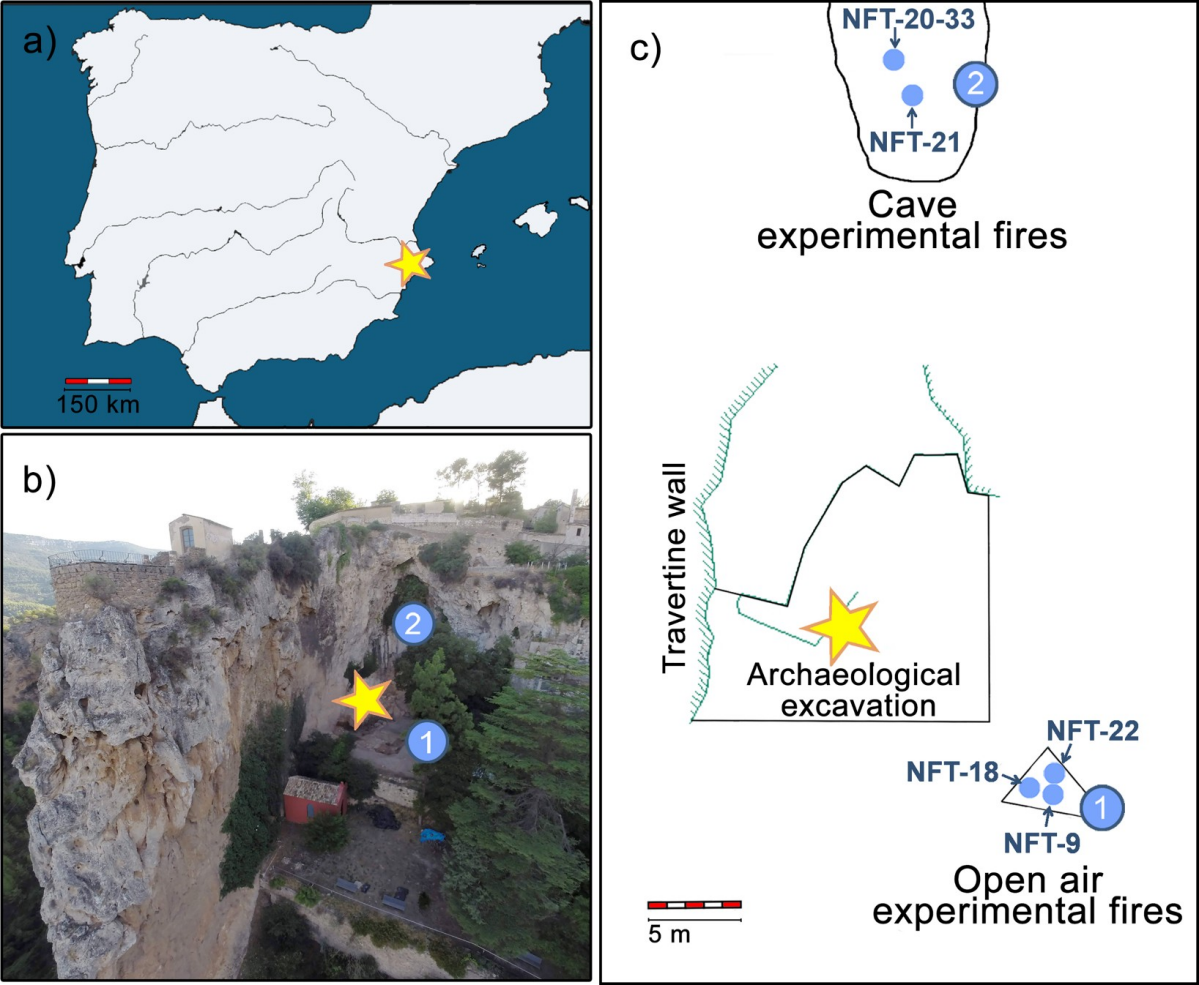
Context and location of the combustion structures. (A) Map of Iberia with the location of El Salt site. (B) Aerial photograph from El Salt. (C) Plan view showing the location of the experimental hearths studied and the archaeological excavation. The yellow star represents the archaeological excavation area. (1) Location of open-air combustion structures NFT-9, NFT-18 and NFT-22. (2) Cave entrance setting in which NFT-20-33 and NFT-21 were made.

Five experimental combustion structures were selected from a larger set of the Neanderthal Fire Technology Project. This project consisted of experimental replication of archaeological combustion structures documented at El Salt Middle Palaeolithic site and the experiments were carried out in the immediate surroundings of this archaeological site (Alcoy, SE Spain; Lat.: 34° 41’ 13” N, Long.: 0° 30’ 32” W; Fig 1). The main variables subject to control were: substrate type, fuel type and quantity, temperatures reached, combustion duration, extinction actions, pre- and post-combustion conditions and time between combustion and sampling (Table 1). Some combustion structures were excavated just after the burning event, while others were abandoned in situ during variable periods of time and underwent diverse taphonomic processes (Table 1). The selected combustion structures are: NFT-9, NFT-18, NFT-20/33, NFT-21 and NFT-22 (S1 Fig). Three of them are open-air combustion structures whereas the two others were carried out inside a cave.

**Table 1:** Combustion structure information.

Combustion structure	Location	Substrate	Fuel	Total amount of fuel (kg)*	Duration of the fire (hours)	Maximum temperatures reached**	Pre-combustion actions***	Actions during combustion****	Extinction	Post-combustion conditions*****	Year of burning	Year of sampling
NFT-9	Open air	Dry unvegetated sediment, with loose silt on the surface. Angular limestone fragments (1-3cm) are common. Isolated grass stalks.	*Pinus nigra* and ivy	16.3	3–4	No availa-ble	Addition of 3 owl pellets (*Bubo bubo*)	Refuel and addition of 0.5 kg of cooked chicken bones, 2 eggs and 2 chopped rabbits (later for consumption)	Natural	Addition of a carbonized horse epiphysis from combustion event 4 of NFT-10-6 and trampling during 16 days.	2010	2015
NFT-18	Open air	Dry sediment, with loose silt on the surface. Angular limestone fragments (1-3cm) are common. Isolated grass stalks.	*Pinus nigra*	23.5	2–3	622°C 663°C	Addition of bone fragments (unrecorded type and amount), travertine and limestone fragments, flint flakes (Mariola-Benimartxó flint)	Refuel and addition of cow bone	Natural	-	2010	2015
NFT-20	Cave	Cemented carbonate-rich silt at cave entrance (carbonate crust). Underlying, there is a detrital layer (with differential thickness along the substrate) (S2 Fig).	*Pinus nigra*	12	3–4	733°C 770°C	Addition of 11 flint flakes (Serreta-Frare Biar flint)	Addition of dry horse excre-ments	Natural	-	2010	2015
NFT-33 (relighting over NFT-20)	Cave	Cemented carbonate-rich silt at cave entrance (carbonate crust). Underlying, there is a detrital layer (with differential thickness along the substrate) (S2 Fig).	*Pinus syl-ves-tris*	-	-	101.6°C 592.4°C 864.4°C	-	-	Natural	-	2013	2015
NFT-21	Cave	Cemented carbonate-rich silt at cave entrance, including some oncolites (S3 Fig)	*Pinus nigra*	7.2	2–3	53°C 93°C 260°C 508°C 706°C 763°C	Addition of limestone and conglomerate cobbles.	Refuel and addition of 2 kg of leftover charred large branches, 1 fragmented horse tibia (distal and proximal ends) and 9 horse ribs	With sediment	-	2010	2015
NFT-22	Open air	Dry sediment, with loose silt on the surface. Angular limestone fragments (1-3cm) are common. Isolated grass stalks.	*Pinus nigra*	19.5	5–6	218°C 420°C 617°C 791°C 868°C	-	Refuel	Natural	-	2010	2015

(*) It includes the total fuel mass: initial amount + refueling during combustion.

(**) Each value corresponds to a different thermocouple; more information in S1 Text.

(***) Although not considered as “pre-combustion action”, an initial amount of fuel was present in all the combustion structures.

(****) Any action performed while the fire was lit.

(*****) All intentional actions performed on the fire after smouldering.

No specific permissions were required for the experimental work presented here except for the making of fires, which was authorized by the Cultural Heritage Department of the Valencia Government. Experimental actions were specifically approved as part of obtaining the field permit. The flint used is not archaeological and was legally collected from local outcrops. The chicken, pig and rabbit bone were not archaeological and were purchased in local supermarkets. No endangered or protected species were involved in this study.

### 2.1 Combustion and post-combustion variables

Combustion and post-combustion variables are compiled in Table 1. Specific details are included in S1 Text.

### 2.2. Taphonomic processes

Different taphonomic processes were observed throughout the time (between two and five years) that the fires were left on the surface. Around 2 months after the firing, open-air combustion structures were covered by leaves from the surrounding trees (*Celtis sp*.). In the following Spring, grass and dry leaves covered the entire area and burrowing insects were present. The first step of the excavation process in 2015 involved careful removal of this grass cover (S4 Fig). Underneath, we found a very loose sedimentary layer composed of grayish-brown aggregates, decayed *Celtis* leaves, decayed *Celtis* seeds and their calcitic seedcoats and charcoal fragments. The cave entrance combustion structures did not undergo any of the mentioned processes. Macroscopically, they seemed intact, with a few small carnivore excrements and footprints on the ash surface and spider webs around the residual logs.

No further disturbance was observed. No new sedimentary surface deposits were noted and the larger combustion residues (calcined bone and limestone cobbles) were found in their primary depositional position.

### 2.3. Combustion structure sedimentary facies

The experimental combustion structures were stratigraphically described in the field upon excavation, which took place two (NFT-33) to five (NFT-9, -18, -21 and -22) years after they were made.

From top to bottom, the following facies were documented in the combustion structures:

- A white and/or grey ash layer (WL). Identification and isolation of ashes during excavation was not always straightforward. Ash from the cave entrance combustion structures was very well preserved, while ash in the open-air combustion structures appeared physically reworked as compact aggregates or mixed with sediment from the underlying layer, possibly as a result of bioturbation.
- A 2–4 cm thick black layer (BL) in the open-air structures. This constitutes the soil substrate on which the fire was performed. Open air combustion structures were developed over organic-rich sediment and BL black colour is possibly due to charring of the organic matter in the soil [32]. The BL of one open-air structure with strong bioturbation by plants was diffuse and difficult to delimit macroscopically: NFT-22. Occasionally, reddened patches appeared (rubifaction) within the BLs. Just below the BLs, progressive colour grading from dark to pale brown was reported.

The two cave structures were carried out over a mainly inorganic substrate (cemented carbonate-rich silt, forming a carbonate crust of *ca*. 2 mm at the top). In the cave NFT-21 combustion structure, oncolites were detected just below the carbonate crust (S3 Fig). In the NFT-20-33 cave structure, a centimeter-thick detrital layer was observed underlying the carbonate crust (S2 Fig). As expected for inorganic substrates [32], the cave entrance structures yielded very thin (1–2 mm) BLs or none. Instead, the surface of the substrate showed darkened or reddened patches.

### 2.4. Working hypotheses and expected results

Heating may have triggered two main magnetic changes in the sedimentary substrate of our combustion structures. Firstly, it is well known that even mild heating induces chemical changes in the magnetic mineralogy (phase transformations or grain volume changes). Depending on the burning conditions, generation of new ferrimagnetic (*s*.*s*.) minerals such as magnetite and/or maghemite, is common in a wide range of burnt materials, including soils and sediments. This is usually identified through a distinguishable magnetic enhancement in the magnetic concentration-dependent parameters as, for instance, magnetic susceptibility or isothermal remanent magnetization (IRM), in comparison with the unburnt surrounding context (e.g.: [76]). This contrast in the magnetic signal produced by mineralogical transformations, gives magnetic methods a high capacity to identify and characterize burning features.

Chemical changes might be interpreted in terms of maximum temperatures reached by the burnt materials. The hypothesis is that if chemical changes (e.g.: neoformation of magnetite) are observed when heating a sample in the lab in thermal experiments the temperature reached by the sample in the field was likely lower than the maximum temperature applied in the lab [55, 63]. It is because the sample is not thermochemically stabilized; otherwise, it should not exhibit mineralogical transformations. This obviously does not always work [81] but we wanted to test this application of the method on these experimental fires and see its viability.

Secondly, physical changes are also expected due to heating. These manifest as new paleomagnetic components. Depending on the heating temperature and the mineralogical changes involved, the direction of the Earth's magnetic field (EMF) can be recorded through different types of magnetization mechanisms. In burnt archaeological materials like these, the most common is known as thermal remanent magnetization or TRM. When a material is heated over the *Curie temperature* (henceforth T_C_) of its main magnetic carrier (T_C_ is specific of every ferromagnetic mineral; e.g.: magnetite T_C_ = 585°C or haematite T_C_ = 675°C), it loses its ferromagnetic properties and becomes paramagnetic. That is, it does not retain magnetic memory in the absence of magnetic field and basically deletes any previous magnetization. However, it is during the cooling process in the presence of the EMF that when crossing the *blocking temperature* or T_B_ (mineral and grain volume dependent and always lower than T_C_), the material records a remanent magnetization parallel and generally proportional to the EMF. If the material is not reheated later and physically remains *in situ*, the archaeomagnetic direction obtained in the laboratory represents a snapshot and stable record of the EMF at the time of its last heating and subsequent cooling.

This behaviour results in univectorial orthogonal NRM demagnetization diagrams of high intensity and whose specimens exhibit directions quite reproducible among them. If the material is not preserved *in situ*, the orthogonal NRM demagnetization diagrams will be characterized by being multicomponent (various vectors), with anomalous directions and weaker intensities of magnetization. Depending on the mineralogical changes involved and when they occur, burnt archaeological materials can record other magnetization mechanisms such as, for example, the thermo-chemical remanent magnetization (e.g.: 76). Likewise, these physical and chemical transformations can be identified through the magnetic properties (composition, concentration and granulometry of ferromagnetic minerals). That is why so important to combine the directional results with the magnetic properties analyzed case by case.

Taphonomic processes may affect the preservation of the magnetic record of archaeological combustion structures as we want to evaluate. In our studied structures, bioturbation is the most common taphonomic process observed. Agents like plants (with their roots going into the ground) and/or animals may cause mechanical processes affecting the burnt facies. Plant growth and soil aggregation as documented in our experimental fires (S1 Text and S4 Fig) may have entailed mechanical reworking, especially within the ashes. A significant scatter in the directions is expected in these cases [30, 55]. Trampling may also affect the directional record. Specifically, flattening is expected as an effect of the pressure exerted on the materials. It can be easily detected in the archaeomagnetic directional record as an inclination shallowing (lower inclinations than the expected).

### 2.5. Sampling

In the field, fourteen oriented hand-blocks (samples) were collected from all combustion structures. Ashes were not sampled given their high volatility and propensity to be remobilized. Archaeomagnetic directional analyses require collection of magnetically oriented samples from *in situ* (*s*.*s*.) burnt materials. As we could not guarantee that these ashes fulfilled this requirement they were excluded. Nonetheless, bulk (unoriented) ash samples from each combustion structure were collected to study their magnetic properties.

After carefully removing the ash facies from each combustion structure, samples were taken by means of oriented hand blocks focusing on the top 2 cm of the thermally altered substrate (BL of open-air structures and darkened carbonate crust of cave entrance structures). Bulk (unoriented) sediment samples of each facies were also collected.

The hand-blocks were sampled by dripping a creamy-textured mix of plaster of Paris and water on them, gently pressing a piece of methacrylate on the plaster while wet, levelling it using a bubble level and allowing it to set. Upon drying, orientation was recorded using a magnetic compass. No more than two hand-blocks per combustion structure were collected, allowing for other geoarchaeological sampling.

When possible, oriented samples were preferably collected from the central area of the combustion structures (S5 Fig). Part of the uppermost area of one block from cave combustion structure NFT-21 (the most strongly burnt area) broke and was lost during sampling.

Apart from the burnt sediment samples, four unburnt control samples (oriented hand blocks) were collected. These comprised two carbonate crust samples with underlying detrital cave sediment and two samples of soil-sediment from the open-air area.

Blocks from NFT-9 and NFT-22 combustion structures and unburnt control samples from the open-air area were consolidated with sodium silicate (75%) mixed with distilled water (25%). Afterwards, samples were cut into cubic or cylindrical specimens of around 10 cm^3^. The final sample set comprised a selection of 88 specimens. From them, 41 are from the uppermost part of the combustion structure hand blocks (0–2 cm of depth), 26 from the intermediate part (2–4 cm), 5 from the lowest part (4–6 cm) and 16 from the unburnt blocks.

### 2.6. Laboratory methods

All the analyses reported here were carried out in the Laboratory of Palaeomagnetism of Burgos University, Spain. The natural remanent magnetization (NRM) directional stability was analysed in 73 burnt and unburnt (oriented) specimens to assess the directions recorded in the burnt sediments. Stepwise progressive thermal (TH) demagnetization of the NRM was performed in 14–21 steps up to 585–600°C using a TD48-ASC oven and the remanence was measured with a 2G-755 cryogenic magnetometer (noise level: ~ 5 x 10^−12^ Am^2^). Two additional burnt specimens per combustion structure along with other five unburnt specimens were analysed by stepwise alternating field (AF) demagnetization with the automatic demagnetization unit coupled to the 2G magnetometer in 20 steps up to a peak field of 100 mT. Deletion of the natural remanent magnetization by progressive demagnetization steps allowed us to reconstruct the direction of the magnetization vector. The analysis of demagnetization data and the calculation of the characteristic remanent magnetization (ChRM) direction was performed using the software Remasoft 3.0 [82].

The expected Earth´s magnetic field (EMF) directions for the dates and place of the experimental burnings were calculated using the model IGRF v. 12 [83]. The calculated direction for El Salt site between August 6^th^ and 20^th^ of 2010 is: Dec. = 359.3°, Inc. = 53.2° whereas for March 27^th^ of 2013 is: Dec. = 359.6°, Inc. = 53.3°. The angular distance between both directions is negligible for our purposes.

Low-field magnetic susceptibility of all specimens was measured initially at room temperature with a KLY-4 Kappabridge (AGICO, noise level: 3 x 10^−8^ S.I.).

The Koenigsberger ratio (Qn = NRM/(χH), where χ is the magnetic susceptibility and H is the local magnetic field strength [84]) was calculated for the entire collection of oriented samples. This parameter provides a quick estimate of the efficiency of the NRM acquisition mechanism based on the relationship between the induced and the remanent magnetization and shows high values in the case of thermal remanent magnetization (TRM).

With the aid of a Magnetic Measurements Variable Field Translation Balance (MM_VFTB) several rock-magnetic analyses were carried out in order to further constrain the magnetic mineralogy, domain state and thermal stability. These comprised progressive isothermal remanent magnetization (IRM) acquisition curves, hysteresis loops (± 1T), backfield coercivity curves and thermomagnetic curves until 700–800°C in air on bulk (non oriented) sample of each representative burnt facies and unburnt samples. IRM progressive acquisition consisted in applying incremental field steps and measuring the remanence (after removing the field) at each step. This measurement exclusively showed the ferromagnetic contribution and shed light on the coercivity of ferromagnetic minerals present in the studied materials. Backfield involved the same experiment, but applying the field in the opposite sense. This experiment provided a parameter called remanent coercive force (B_CR_). Hysteresis cycles measured the magnetization of the sample submitted to cyclic applied magnetic fields up to ± 1 T. Hysteresis showed the contribution of both ferromagnetic and non-ferromagnetic fractions (dia- and paramagnetic minerals). After correcting the dia/paramagnetic fraction, hysteresis parameter ratios (remanent saturation to saturation magnetization (M_RS_/M_S_) and remanent coercive force to coercive force (B_CR_/B_C_)) were calculated for the sample set and displayed in the so-called “Day plot” [85,86]. Day plot shows the dominant domain state of ferromagnetic minerals in samples in which magnetite is the main magnetic carrier. It is basically related to the ferromagnetic grain size and how stable the magnetic signal is. The magnetization dependence with temperature (thermomagnetic curves) was measured in a constant field (around 36 mT) to identify the main magnetic carriers through the estimation of their Curie temperatures as well as to evaluate their magnetic stability. Thermomagnetic curves were systematically measured up to 700°C. Additionally, in order to determine maximum temperatures reached by BL using this experiment as paleotemperature indicator, representative samples from some combustion structures were selected to carry out partial thermomagnetic curves at specific temperature steps. This consists of heating until a maximum temperature of 200°C and subsequently cooling to room temperature, repeating this cycle of heating and cooling in incremental steps of 100°C until 700°C. The software *RockMag Analyzer* [87] was used to interpret all these data.

## 3. Results

### 3.1. Magnetic properties

#### 3.1.1. Unburnt materials

**Fig 2:**
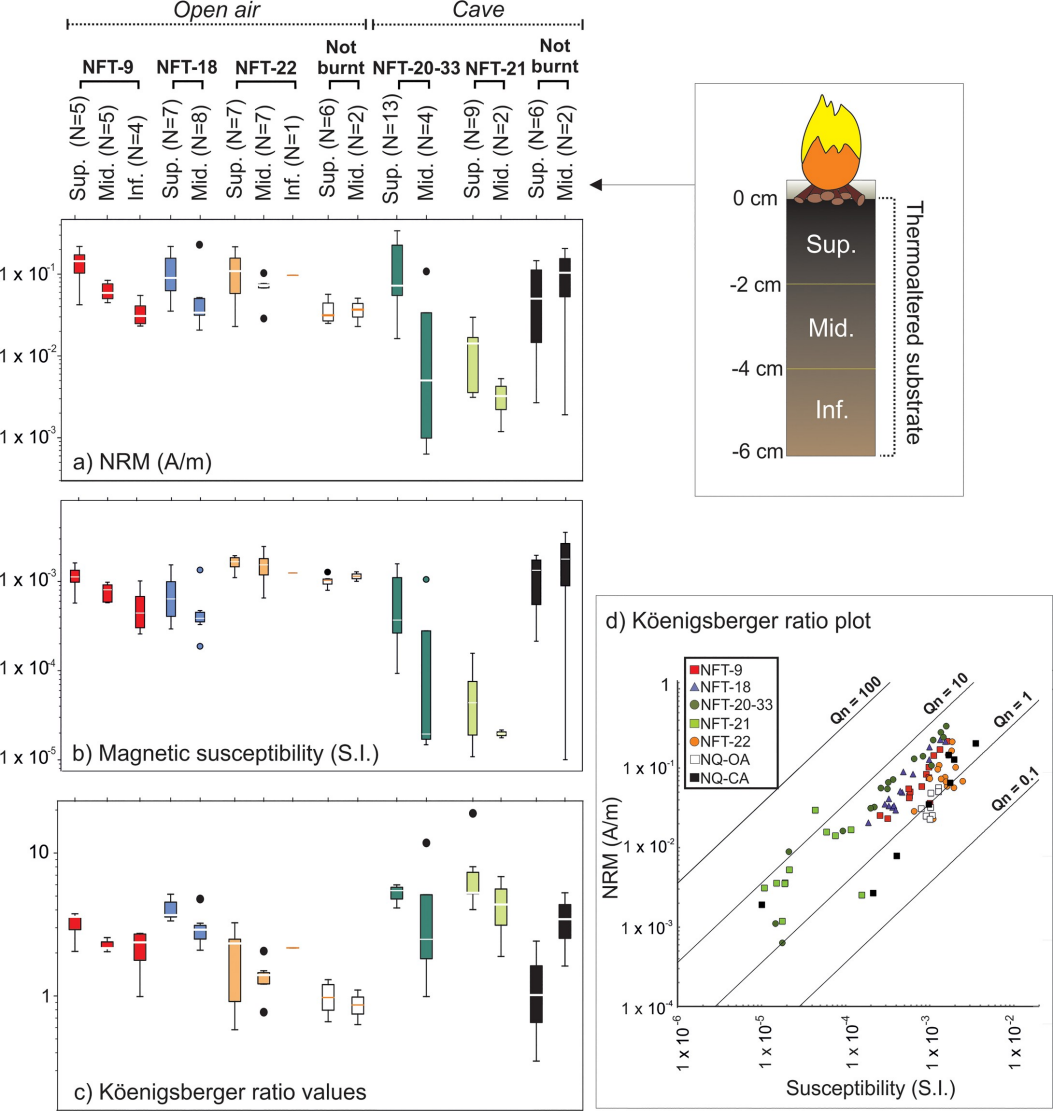
Values of natural remanent magnetization (NRM), initial magnetic susceptibility and Köenigsberger ratio. Box & whiskers plots of (A) natural remanent magnetization intensity (NRM), (B) initial magnetic susceptibility and (C) Köenisberger ratio values per combustion structures/contexts and depth. The line dividing the box corresponds to the median. Black points represent outliers. (D) Köenisberger ratio diagram showing magnetic susceptibility values in axis X, NRM values in axis Y and Köenigsberger ratio values delimited by isolines. All graphics are shown on logarithmic scale.

NRM intensity values of oriented specimens range from 2 x 10^−3^ to 2 x 10^−1^ A/m for the cave combustion structures and from 2 x 10^−2^ to 6 x 10^−2^ A/m for the open-air area (Fig 2A). Cave specimens exhibit susceptibility values between 1 x 10^−5^ and 4 x 10^−3^ (S.I.), whereas open air unburnt specimens range from 8 x 10^−4^ to 1 x 10^−3^ (S.I.) (Fig 2B). Köenigsberger ratio values for the open air materials tend to be slightly lower than those from the cave (Fig 2C and 2D), but in both cases around the half of the specimens presents values higher than 1.

**Fig 3:**
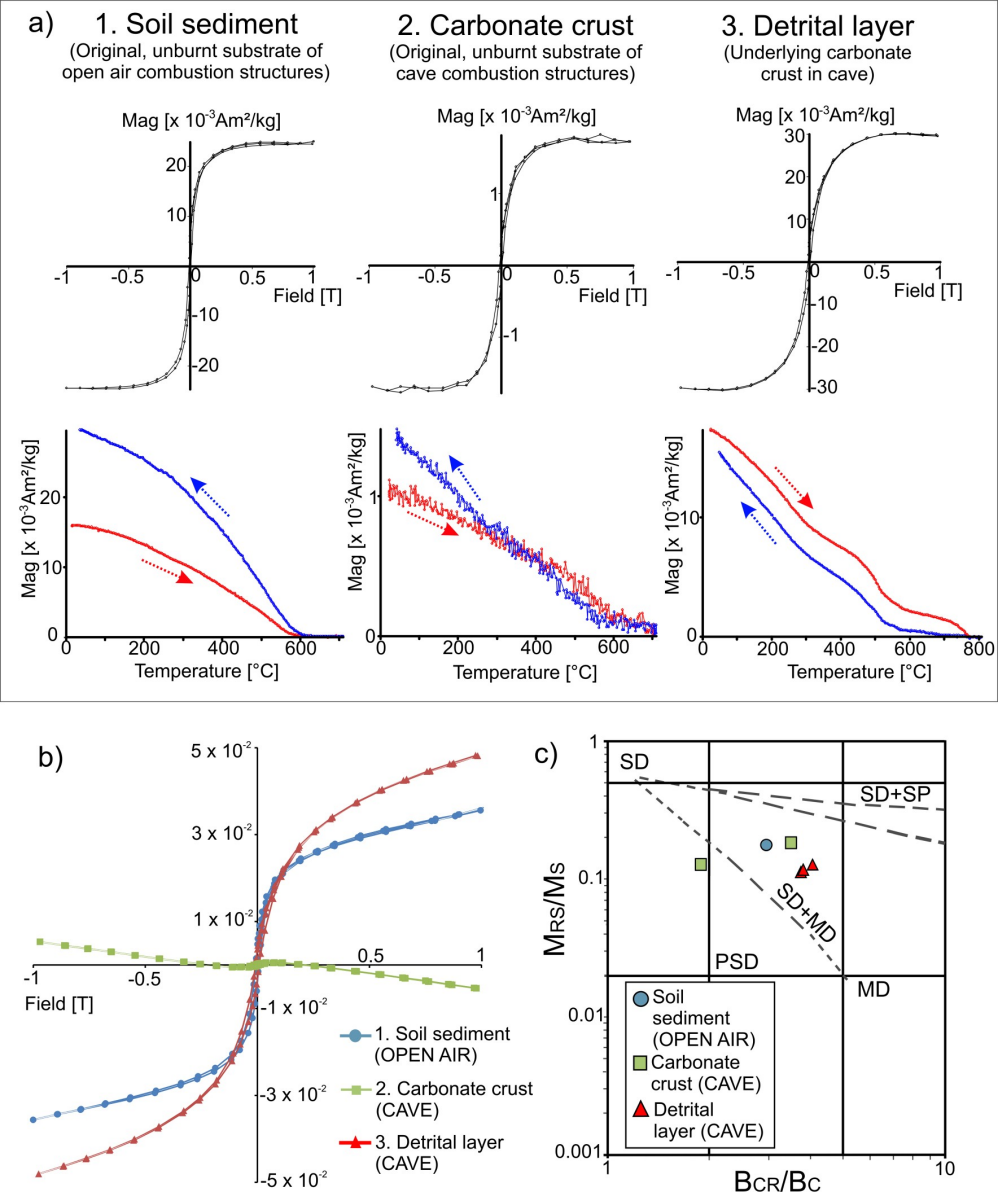
Representative hysteresis loops, temperature-dependent magnetization curves and Day Plot [85, 86] of unburnt materials. (A) Hysteresis loops with para-/diamagnetic correction (up) and thermomagnetic curves with correction of dia/paramagnetic contribution (down) of (1) unburnt open-air substrate and (2) unburnt carbonate crust and (3) detrital layer from the cave. (B) Uncorrected hysteresis plots of the samples showed in (A1), (A2) and (A3). (C) Day plot [85,86] (logarithmic scale) showing representative unburnt samples. The dashed lines represent mixing curves taken from [86] for mixtures of single domain (SD) and multidomain (MD) or superparamagnetic (SP) magnetite grains. PSD refers to pseudo-single domain.

Magnetite seems to be the main ferromagnetic mineral in the unburnt sediment of the open air area and the thermomagnetic curve exhibits a clear irreversible behaviour (Fig 3A.1). In the case of the unburnt carbonate crust main ferromagnetic mineral could not be determined with certainty since thermomagnetic curves are very noisy (Fig 3A.2). Probably magnetite is present. In the case of the detrital layer, magnetite is suggested as the main ferromagnetic mineral, although pure iron (Curie temperature around 770°C) is also detected (Fig 3A.3). After the heating, iron is transformed into haematite and/or magnetite.

The carbonate crust presents lower values of magnetization in the hysteresis plots as well as in thermomagnetic curves, compared with the detrital layer and the open air (soil) sediment (Fig 3A and 3B).

The hysteresis ratios are 0.11 < Mrs/Ms < 0.18 and 1.9 < Bcr/Bc < 4.1, suggesting pseudo-single domain (PSD) behaviour in the Day Plot [85,86] (Fig 3C).

#### 3.1.2. Burnt materials

Initial NRM values of the oriented burnt specimens oscillate between 6 x 10^−4^ and 3 x 10^−1^ A/m (Fig 2A) showing a decreasing intensity pattern with depth. The uppermost specimens (0–2 cm of depth) exhibit higher NRM values than those from the intermediate (2–4 cm) and deepest (4–6 cm) depths both for open-air and cave combustion structures.

The magnetic susceptibility values of the oriented burnt specimens oscillate between 1 x 10^−5^ and 2.5 x 10^−3^ (S.I.) (Fig 2B). Similarly to the NRM, the same decreasing pattern in depth is also observed in this parameter both for cave and open-air combustion structures.

Köenigsberger (Q_n_) ratio values of burnt materials (both open-air and cave combustion structures) are mostly comprised between 1 and 19, suggesting that the magnetization mechanism is consistent with thermal origin (Fig 2C and 2D). As expected, a decrease as a function of depth is observed and unburnt specimens tends to exhibit lower Q_n_ ratio values. Within their variability, Q_n_ ratio values of cave combustion structures are higher than those from open-air combustion structures (Fig 2C), mainly because the low susceptibility of the first.

**Fig 4:**
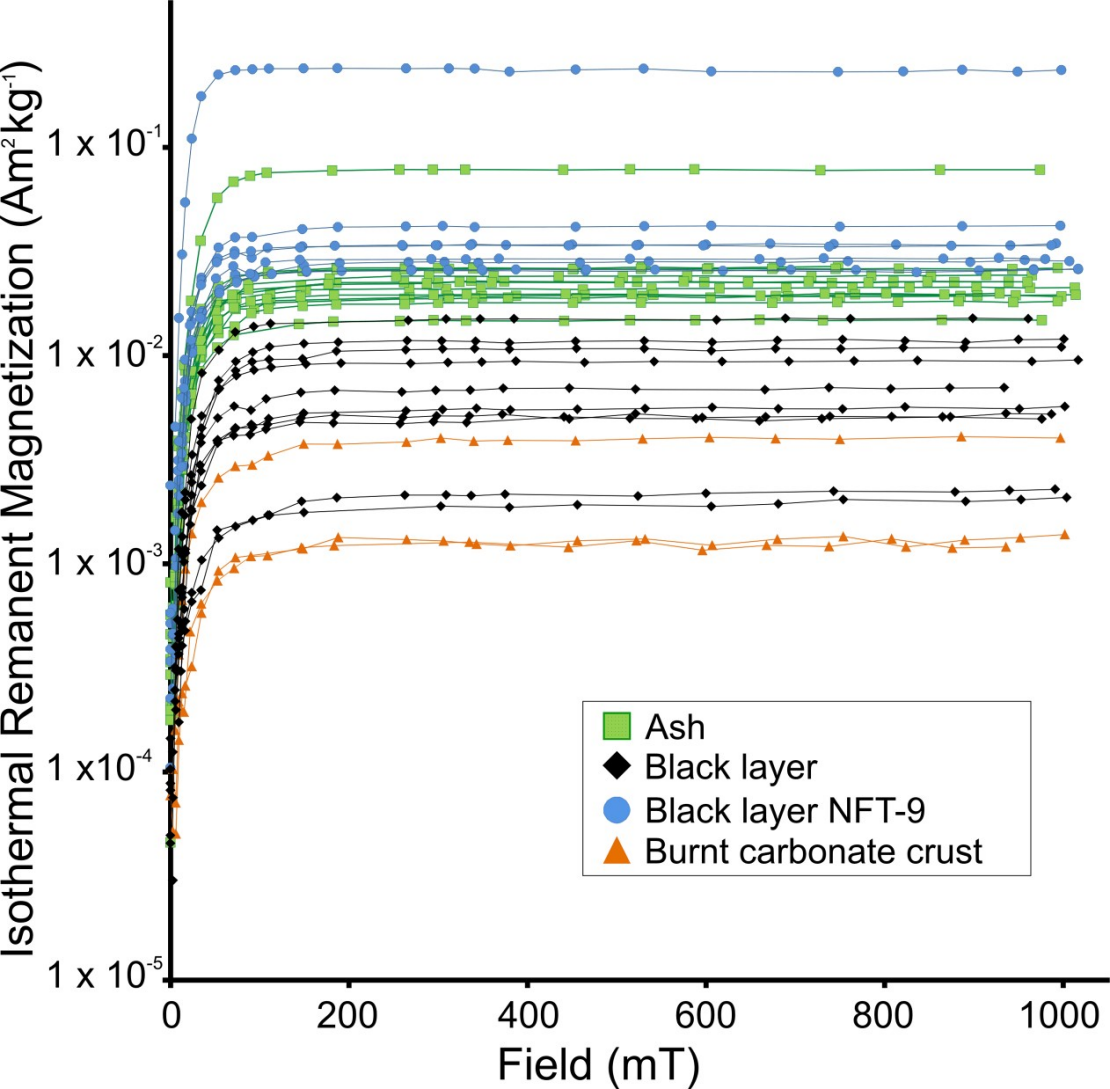
Progressive IRM acquisition curves per facies on logarithmic scale. Green squares = ash; black diamonds = black layer; blue circles = black layer from the intensively trampled NFT-9; orange triangles = burnt carbonate crust). Axis Y is on logarithmic scale.

Progressive IRM acquisition curves show that the main ferromagnetic minerals are low coercivity minerals (e.g.: magnetite and/or maghaemite), because saturation is almost reached between 100–300 mT (Fig 4). The intensity of IRM curves is higher in ashes, followed by black layers and the burnt carbonate crust samples. The only exceptions are the black layer samples of NFT-9. Their values are higher than those of ashes from other combustion structures.

**Fig 5:**
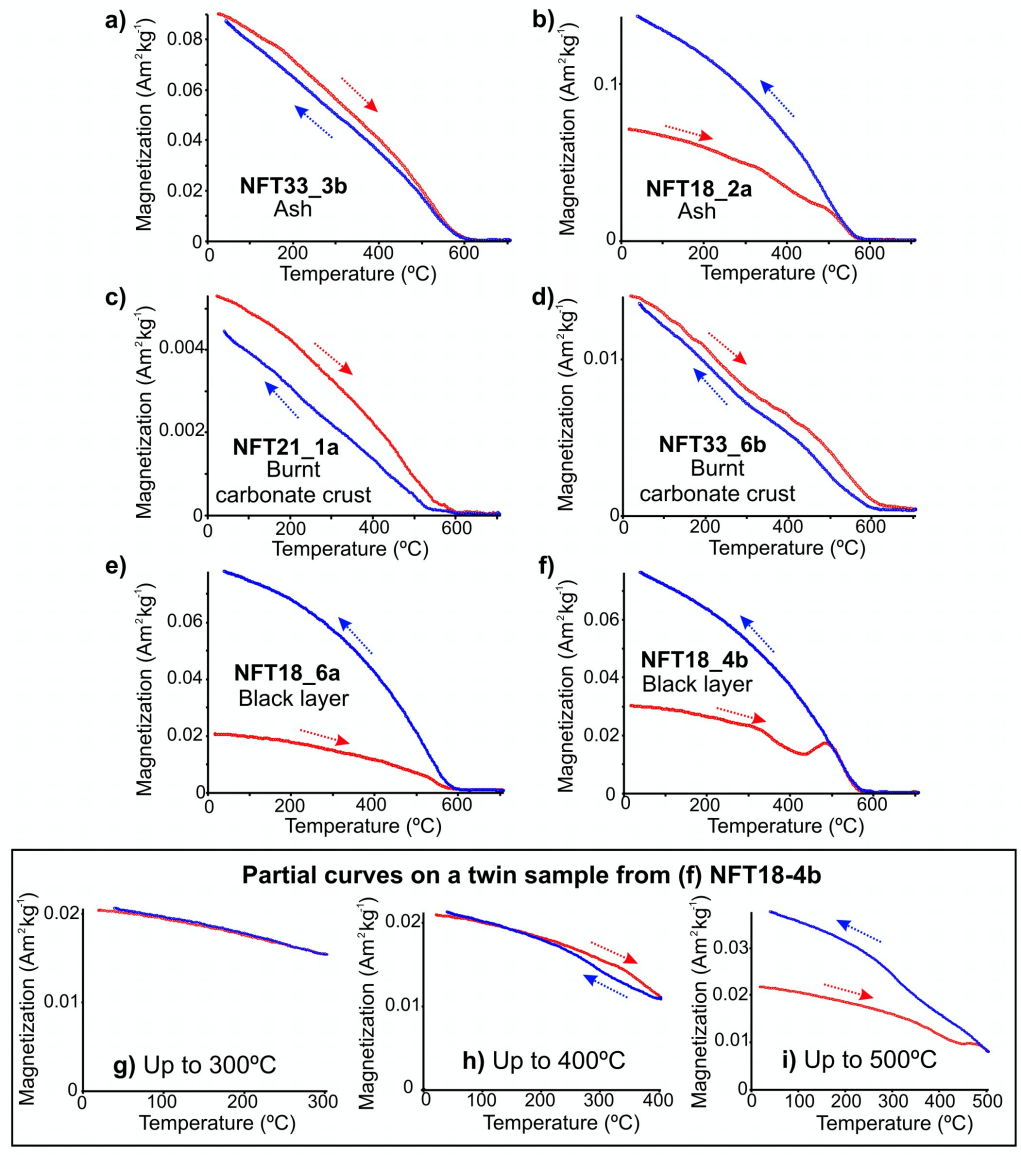
Representative thermomagnetic curves (temperature dependence of magnetization) of burnt materials. (A-B) Thermomagnetic curves of ash samples, (C-D) burnt carbonate crust and (E-F) black layer, all up to 700°C. (G-I) Partial thermomagnetic curves up to 300°C (G), 400°C (H) and 500°C (I) of a twin sample of black layer shown in (F).

Thermomagnetic curves indicate that the main magnetic carrier is magnetite with curie temperatures (T_C_) of around 550–580°C (Fig 5A–5C, 5E and 5F). Occasionally, higher T_C_ up to 600–620°C is observed, suggesting that partially maghemitized magnetite is also present (Fig 5D). The thermomagnetic behaviour of ashes is heterogeneous in spite of being the most magnetic facies. Some samples are highly reversible–heating and cooling cycles coincide- (Fig 5A) while others not, showing also the neoformation of secondary magnetite (Fig 5B). BL curves are always irreversible and less magnetic than ashes (with the exception of the BL of NFT-9), showing a significant increase of magnetization on the cooling cycles (Fig 5E and 5F). In some cases, a new ferromagnetic phase is created starting around from 400–450°C (Fig 5F). It is secondary magnetite. Partial thermomagnetic curves in incremental temperature steps were carried out on a twin sample to that shown in Fig 5F. The heating and cooling cycle up to 300°C is clearly reversible (Fig 5G). The partial run up to 400°C is highly reversible, although slight irreversibility could be suggested (Fig 5H). The cycle up to 500°C is clearly irreversible and secondary magnetite is created (Fig 5I).

Magnetite is the main ferromagnetic mineral in the burnt carbonate crust (Fig 5C). However, in some cases a phase with Curie temperatures slightly above 600°C and an inflection around 300°C also reproducible on cooling is observed (Fig 5D).

**Fig 6:**
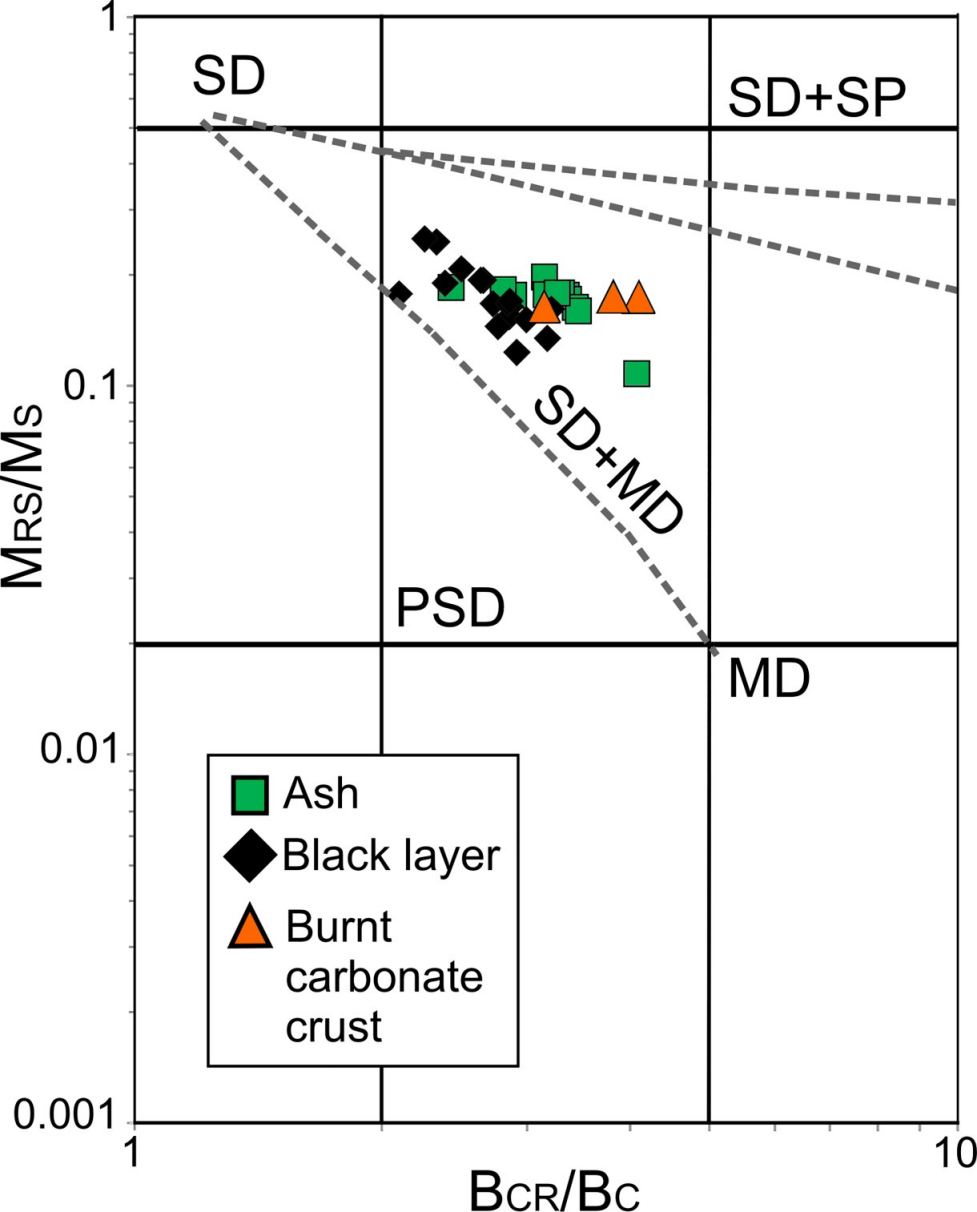
Day plot [85,86] of burnt samples (logarithmic scale). This graphic shows the ratio of saturation remanence (M_RS_) to saturation magnetization (M_S_) *vs*. the ratio of remanent coercive force (B_CR_) to coercive force (B_C_). Regions of single domain (SD), pseudo-single domain (PSD) and multi domain (MD) magnetite particles are plotted according to [86], as well as theoretical mixing curves showed as dashed lines including also SP (superparamagnetic) grains.

The hysteresis ratios obtained range from 0.11< Mrs/Ms < 0.25 and 2.1 < Bcr/Bc < 4.1 for the sample set studied. Analysis of hysteresis parameter ratios pointed towards PSD behaviour in the Day plot (Fig 6).

### 3. 2. Directional stability of NRM

#### 3.2.1. Unburnt control samples

**Fig 7:**
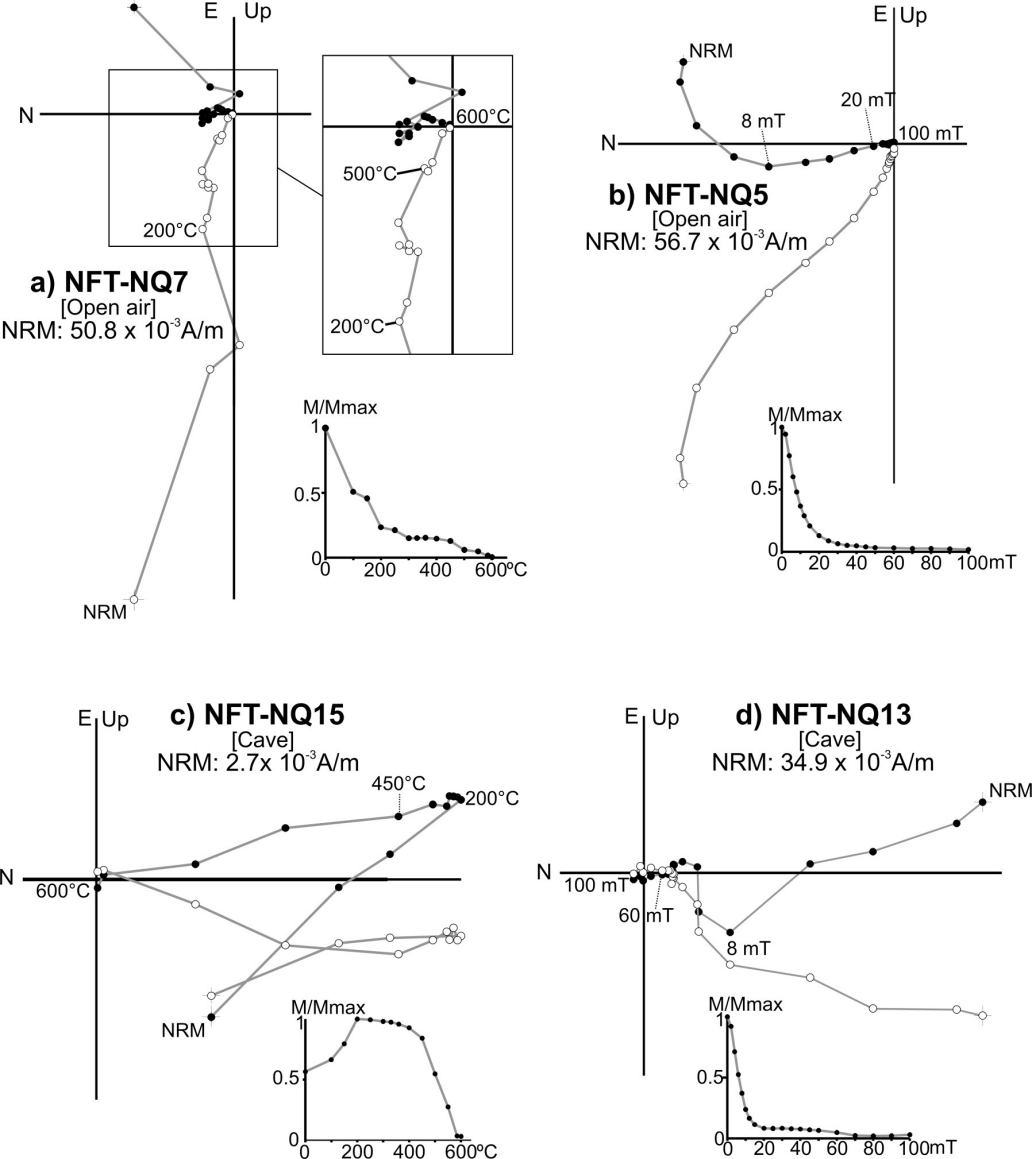
Representative orthogonal plots of NRM thermal and alternating field demagnetizations of samples from unburnt materials. (A-B) Examples from open air; (C-D) examples from cave. This graph shows the structure and direction of the magnetization vector(s) of a sample during the stepwise demagnetization process. Each point represents a demagnetization step. Conventionally, the direction of the vector is defined by two angles: declination (angle respect the North on the horizontal plane) and inclination (angle between the vector and the horizontal plane: downward or upward). Solid (open) circles show projections of vector endpoints onto the horizontal (vertical) plane Sample code, magnetization intensity and normalized NRM intensity decay plots are also shown.

Unburnt materials from both the cave and open-air areas show heterogeneous NRM directional behaviours (Fig 7). Samples exhibit two or more palaeomagnetic components, clearly overlapped in AF diagrams (e.g.: Fig 7B). In the open air, it is most likely due to physical reworking undergone by this soil which was used until recently for gardening purposes. Nevertheless, a northward low-temperature/low-coercivity component is often observed (e.g.: Fig 7A and 7C). It is interpreted as a viscous overprint acquired after the mechanical reworking.

#### 3.2.2. Combustion structures

The lowest-coercivity (< 6–8 mT) or lowest-temperature (< 100–150°C) steps are interpreted as a viscous overprint acquired after heating.

**Fig 8:**
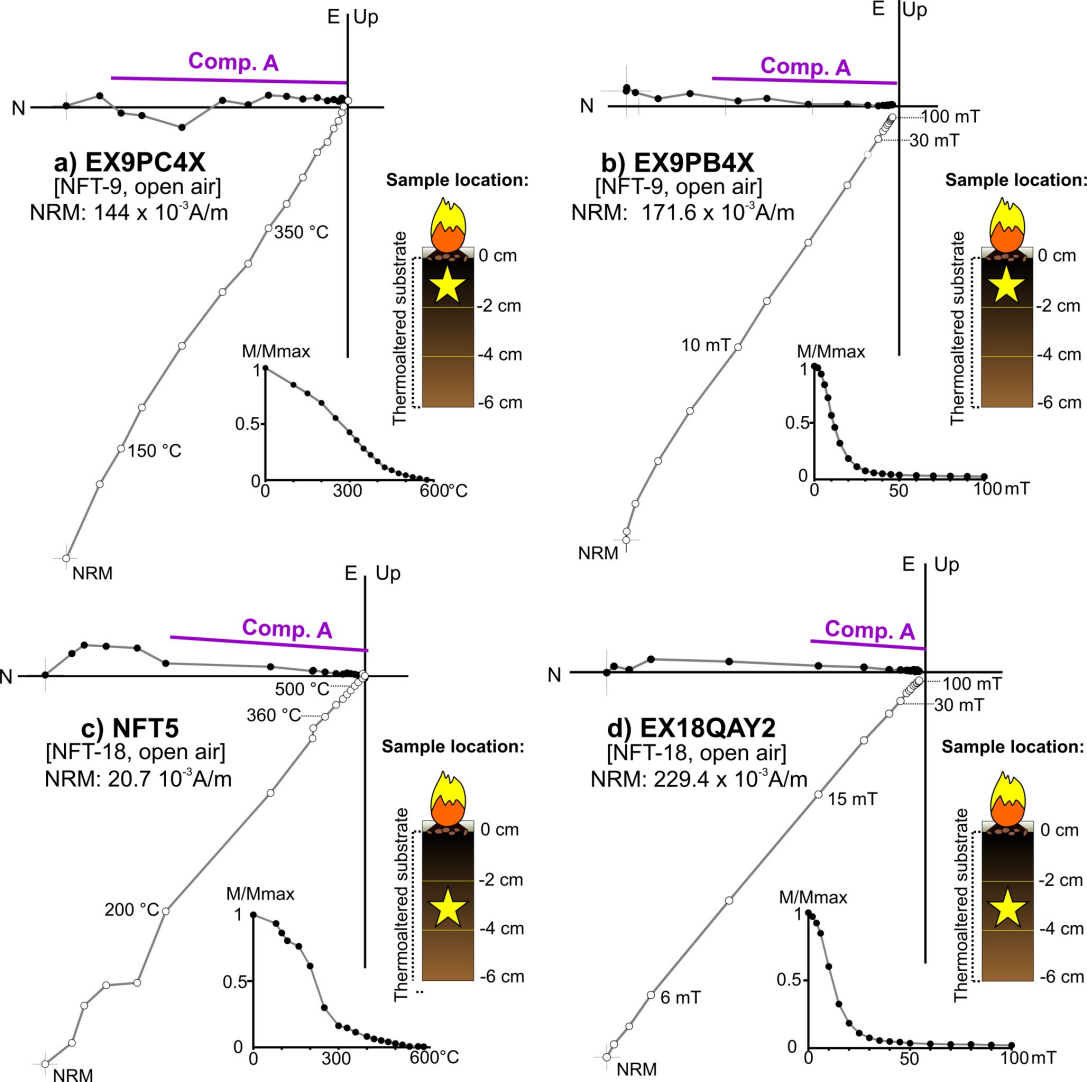
Representative orthogonal NRM thermal and alternating field demagnetizations plots of samples showing component A. (A-B) Superficial specimens; (C-D) intermediate specimens. Symbols as in Fig 7.

**Fig 9:**
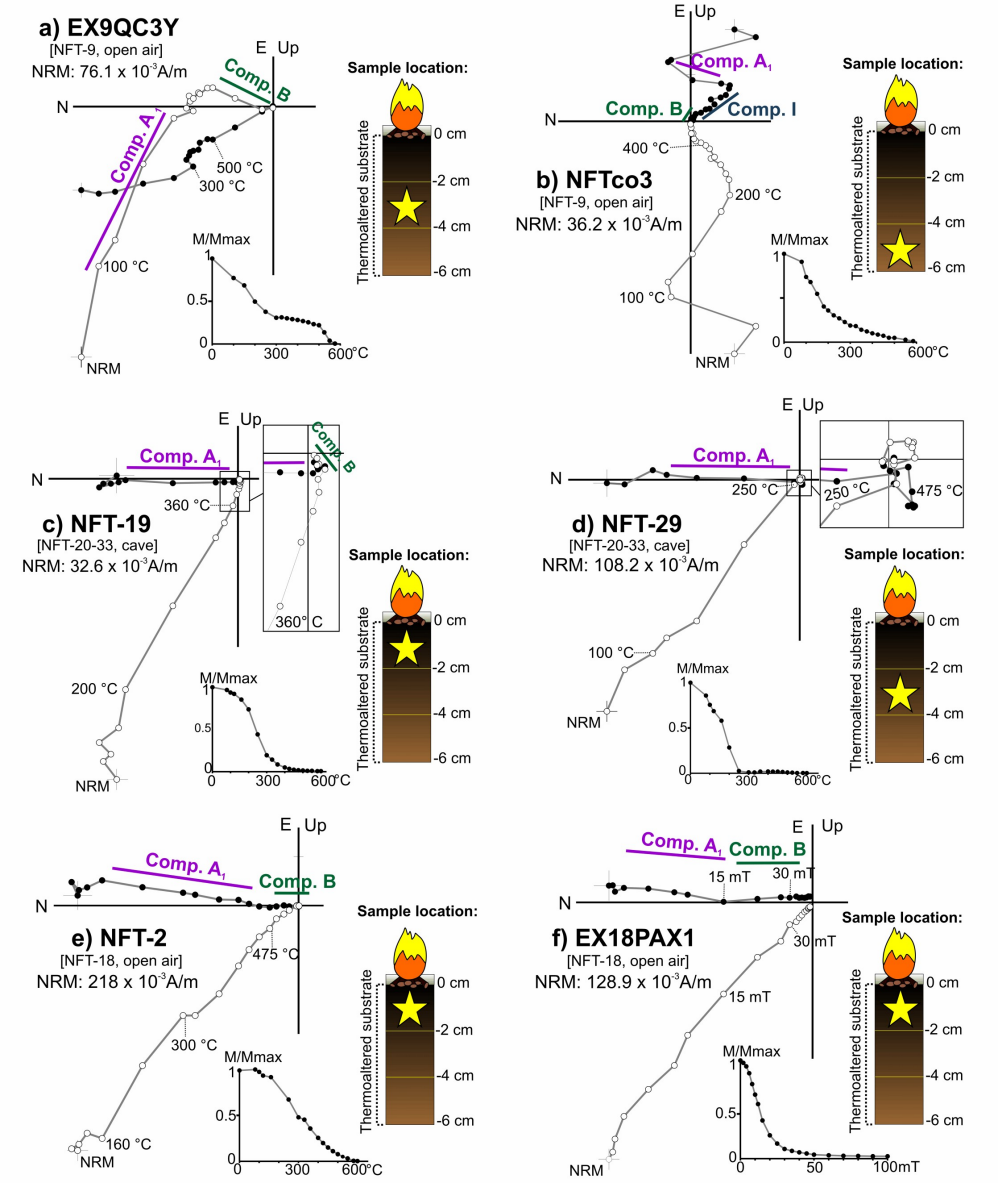
Representative orthogonal NRM thermal and alternating field demagnetizations plots of samples showing component A_1_. Stratigraphic location of each sample with respect to the surface is indicated for each panel. For clarity, the higher temperature steps of panels C and D are blown-up showing the presence of high-temperature components. Symbols as in Fig 7.

**Fig 10:**
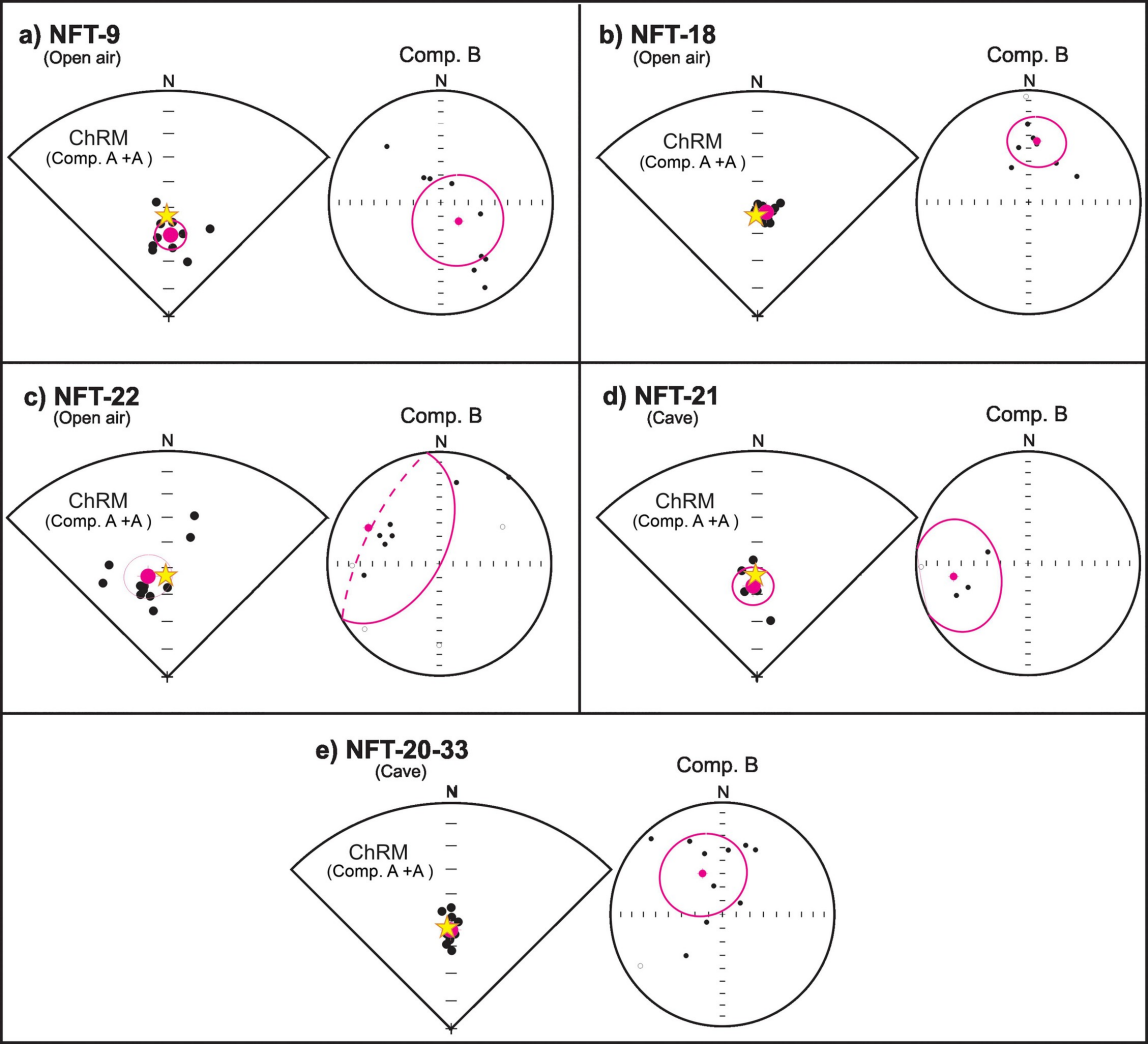
Equal area projections of the mean directions obtained. (A) NFT-9; (B) NFT-18; (C) NFT-20-33; (D) NFT-21; (E) NFT-22. Equal area projection shows the paleomagnetic directions described in the text of every specimen considered reliable (black points) and the mean direction obtained with its respective α95 semi angle of confidence (95% of probability), depicted as pink point and pink circle, respectively. The declination reads clockwise and is represented around the perimeter of the circumference, while the inclination varies from 0° (perimeter) to 90° (center of the circumference). Solid (open) symbols correspond to downward (upward) inclination. In the left part of each panel, ChRM mean direction (components A and A_1_) is shown; in the right part, the mean direction of the high-temperature component (component B). The yellow star is the expected Earth´s magnetic field direction at the time of burning.

**Fig 11:**
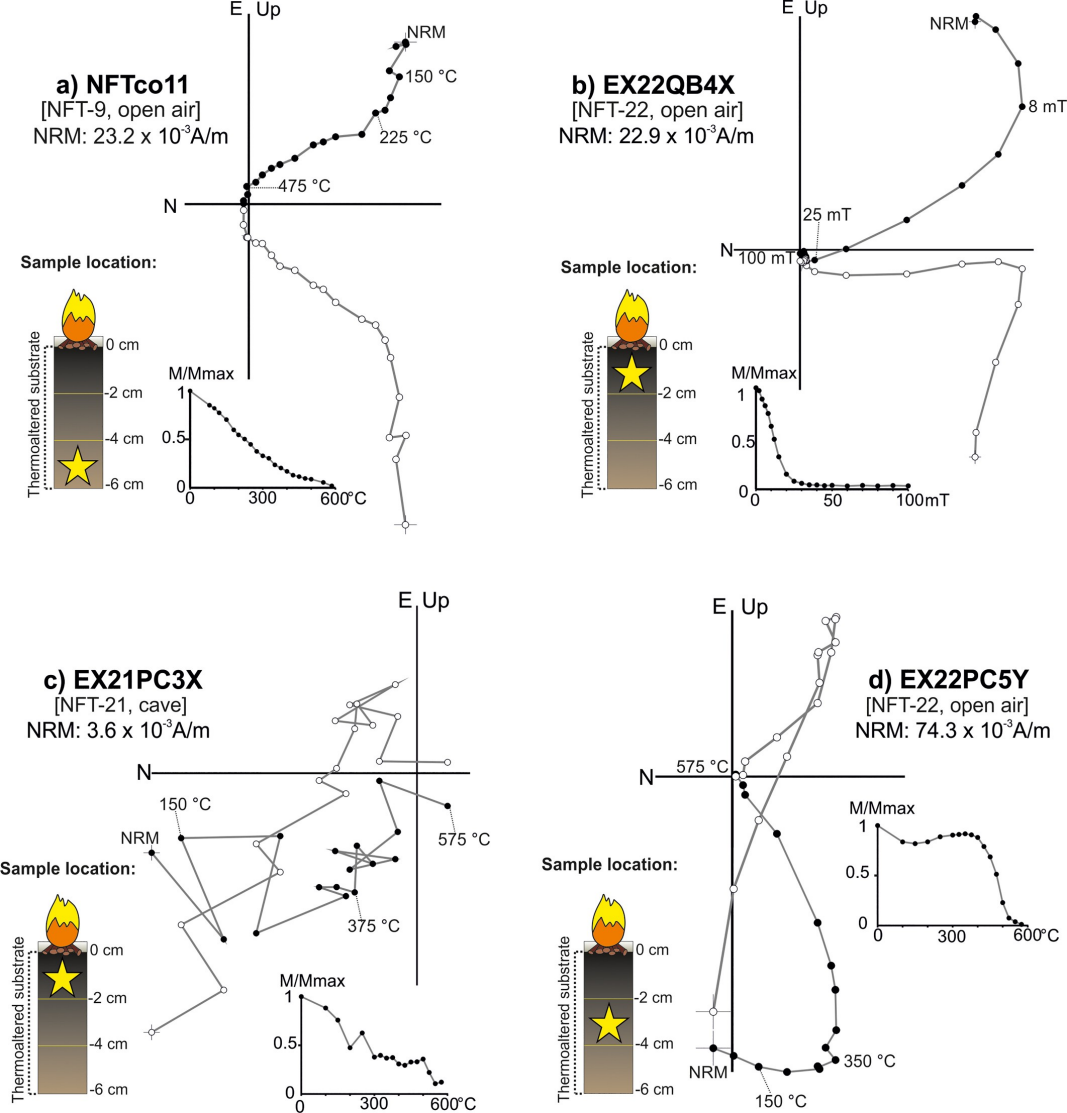
Representative orthogonal NRM thermal and alternating field demagnetizations plots of samples showing anomalous NRM behaviour. Stratigraphic location of each sample with respect to the surface is indicated for each panel. Symbols as in Fig 7.

Three main directional behaviours have been distinguished and described below:

- A single component (A) whose direction is coherent with the local Earth’s magnetic field and interpreted as the record related to the fire (Fig 8A–8D). Despite the NRM orthogonal demagnetization diagrams are defined by a single component, different types of intensity decay curves have been observed among the TH specimens. In some cases, the drop is gradual (Fig 8A), while in others there is a major decay at lower temperatures (*ca*. 250–300°C, e.g.: Fig 8C). AF demagnetized specimens are dominated by low-coercivity minerals (Fig 8B and 8D) with approximately the 90% of the NRM demagnetized at 30 mT and mean destructive fields (MDF) around 15–20 mT.
- A low temperature component (A_1_) of normal polarity interpreted as the record associated to the last heating (Fig 9A–9F). This is the most common behaviour observed. The A_1_ component is detected up to variable temperatures that oscillate between 150 and 500°C. It agrees well with the gradient of temperatures expected for these structures depending on the superficial location of samples (center *vs* periphery) and the depth. Intensity commonly shows a considerable drop at temperatures very close to the maximum unblocking temperatures (max T_UB_) of the component A_1_ (Fig 9A–9D). At higher temperatures, the behaviour is more complex. A high-temperature component (B) (Fig 9A–9F**)** is observed with a scattered direction (Fig 10). It is interpreted as the magnetic record prior to the fire. As we suggested for unburnt materials, mechanical movements suffered by the substrate before the heating seems to be the cause of this dispersion in open area combustion structures. Occasionally, a third component is observed (Fig 9B). The AF demagnetised specimens again show a low coercivity behaviour with almost the 90% of the NRM destroyed at 30 mT (Fig 9F) and MDFs about 15–20 mT.
- Samples showing unstable and erratic NRM behaviour, anomalous directions and/or low intensities of NRM, which does not allow to isolate the ChRM (Fig 11). It is interpreted as the result of a very low (or even absence of) thermal impact. The specimens exhibiting this pattern and those broken during the demagnetization were excluded in the calculation of the mean ChRM directions.

In the cave combustion structures, the most common pattern in this third category is an erratic and unstable diagram with low NRM values (e.g.: Fig 11C). This is logical since in absence of heating the ferromagnetic content in carbonate crust is very poor. In the open air context, samples with moderate higher NRM values are observed but the diagrams show clear overlappings (curvatures) and anomalous directions (Fig 11A and 11B).

Fig 10 illustrates the mean directions of the ChRM (comps. A and A_1_) of each combustion structure and their corresponding statistical parameters are shown in Table 2. The angle between the calculated and the expected mean direction (β) is also included in that table.

**Table 2:** Summary of the statistical parameters associated to the ChRM mean directions (components A and A_1_). From left to right: name of the combustion structure, N’/N (number of specimens used to calculate the direction/total number of demagnetized specimens), declination, inclination, parameter of precision *k*, α95 confidence cone error and β (angle between the calculated and the expected direction).

Hearth	N/N’	Dec. (°)	Inc. (°)	*k*	α95 (°)	β (°)
NFT-9	10/14	1.4	60.4	79.5	5.5	7.3
NFT-18	14/15	4.8	51.9	565.3	1.7	3.6
NFT-20-33	14/17	359.7	53.9	288.1	2.3	0.6
NFT-21	6/11	357.5	56.9	89.4	7.1	3.8
NFT-22	11/15	349.2	52.6	31.8	8.2	6.1

## 4. Discussion

The magnetic signature of burnt materials is affected by three main factors: (1) pre-burning conditions, (2) burning and (3) taphonomic processes. In order to detect and isolate the effects of taphonomic processes in the magnetic signature, the effects of pre-combustion conditions and burning should also be assessed. In the following sections we discuss the results of rock magnetic properties and NRM features in our experimental combustion structures and unburnt sediment in relation to the three issues mentioned above.

### 4.1. The influence of the substrate in the magnetic signature

The nature of the original (unburnt) substrate has a clear influence in the resulting magnetic values of the combustion substrate facies (BL and burnt carbonate crust). The substrate of open-air combustion structures contains organic matter and is rich in ferromagnetic minerals (*s*.*l*.) as indicated by concentration-dependent parameters such as magnetic susceptibility (Fig 2B). The presence of ferromagnetic minerals such as magnetite in soils is rather common. These pre-combustion conditions of the substrate result in little change in concentration-dependent parameters upon heating (Fig 2B). Despite the high temperatures reached in these open-air combustion structures, ferrimagnetic neoformation was limited. In unburnt cave materials, magnetization values of the carbonate crust in the hysteresis plots and thermomagnetic curves are lower than those of the detrital layer underlying the carbonate crust. (Fig 3A and 3B). This is interpreted as a difference in the presence of ferromagnetic minerals (*s*.*l*.). The carbonate crust is considerably poorer in ferromagnetic minerals than the detrital layer. Therefore, the proportion of detrital layer versus the carbonate crust included in each sample influences the results. Samples used for rock magnetic analyses (~300 mg) allowed us to isolate every facies in detail. However, the oriented cubes (~ 8–10 cm^3^) include both facies and cannot be separated. The high dispersion of NRM and magnetic susceptibility values of unburnt oriented specimens is probably an effect of the variability in the proportion of detrital layer, which is different for each specimen. For this reason, the comparison of NRM and susceptibility values between unburnt and burnt sediment must be interpreted with caution.

The substrate also influences the Q_n_ ratio. As expected, unburnt samples exhibit lower Q_n_ values than the burnt samples in their respective contexts. Some cave sediment samples show high Q_n_ values even when NRM values are not very high (see the results for intermediate (2–4 cm) unburnt cave samples, Fig 2C). In these cases, Qn ratio is affected by low susceptibility values due to the influence of the diamagnetic fraction (which strongly reduces the susceptibility).

The origin of the iron identified in the detrital layer is for the moment unknown. However, its presence in the unburnt substrate before heating does not compromise the magnetization record produced by fire.

### 4.2. Variation of the magnetic signature by heating

As far as the magnetic composition is concerned, all the samples (open-air, cave entrance and unburnt sediment) are dominated by low-coercivity minerals, mainly magnetite. The Day plot shows that all the samples are quite well clustered in the pseudo-single domain (PSD) area, which suggests that heating conditions homogenize the magnetic granulometry of the burnt facies (Fig 6). However, interesting differences among burnt facies were observed. The most strongly burnt facies (ashes) display the highest IRM values followed in decreasing order by black layer and carbonate crust samples (Fig 4). As all samples are dominated by the same ferromagnetic mineralogy (mainly magnetite), IRM can be interpreted as a magnetic concentration-dependent parameter (higher magnetization equates to higher concentration of ferromagnetic minerals). IRM points to a higher concentration of ferromagnetic minerals in ashes than in the BL and burnt carbonate crust samples. An exception is given by the BL samples from the NFT-9 open-air combustion structure, which displays higher values than ashes from other combustion structures. Unfortunately, it was not possible to isolate pure ash from the NFT-9 combustion structure in the field for rock-magnetic analysis because this combustion structure was intensively trampled for two weeks. Most probably, these values are related to taphonomic processes as will discussed in section 4.3.

A variable heat impact in the combustion substrate layers (BL and burnt carbonate crust) is reflected in the decreasing NRM intensity and magnetic susceptibility with depth (Fig 2A and 2B).

Thermomagnetic curves suggest that BLs reached temperatures clearly lower than 700°C, since thermomagnetic curves up to such temperature are always irreversible (heating and cooling does not coincide) (Fig 5E and 5F) and show neo-formation of magnetite. Otherwise, they should exhibit thermomagnetic reversibility when re-heated to that temperature in the lab. This behaviour has been previously observed in similar burnt cave facies [55,88,89]. Partial thermomagnetic curves were carried out on a twin sample of one of the samples showing irreversible behaviour in the thermomagnetic curve up to 700°C (Fig 5F). The change from a highly reversible behaviour in the partial curve up to 400°C to clearly irreversible behaviour in the curve up to 500°C (Fig 5H and 5I) suggests that this sample was not originally heated over 500°C (probably 450°C, considering the increasing of magnetization during lab heating around this temperature in the curve up to 700° C, Fig 5F). These results agree with the pTRMs identified in the directional analyses, which are characterized by maximum unblocking temperatures of up to 500° C. Moreover, it is also coherent with the interpretation of BLs given by Mallol *et al*. [32] according to which the colour of BLs results from incomplete combustion of organic matter contained in the original substrate.

The different behaviours observed in the orthogonal NRM demagnetization diagrams of the combustion substrate facies (BL and burnt carbonate crust) are interpreted in terms of distinct magnetization mechanisms related with heating impact. Component A is interpreted as a TRM when in addition to an univectorial structure of the NRM, the intensity decay curves are gradual (Fig 8A). However, in cases in which the intensity drop is more abrupt at intermediate temperatures in the intensity decay curves (*ca*. 300°C; e.g.: Fig 8C), TCRM is suggested as the magnetization mechanism. This interpretation would imply that these samples did not exceed the Curie temperature (T_C_) of the main magnetic carrier (magnetite T_C_ = 585°C;). The first part of the component (up to the temperature at which the abrupt drop is detected; *ca*. 300°C) contains magnetic grains with T_B_ below the maximum heating temperature reached. The magnetization mechanism of this part is a thermal magnetic remanence. The remaining steps include the new minerals created by fire but with T_B_ higher than the maximum temperature reached. In this case, those grains are of chemical origin. As both parts are acquired in presence of the same field, they are not directionally distinguishable and the directional record is reliable [90]. Carrancho and Villalaín [76] demonstrated this hypothesis in a study of an experimental combustion structure made on a clayey substrate.

Component A_1_ is interpreted as a pTRM related to the fire and component B is interpreted as a record prior to the fire (Fig 9). Therefore, these samples did not exceed the Curie temperature or the main remanence carrier. It is worth mentioning that maximum unblocking (max. T_UB_) temperatures of the pTRMs are coherent with the results of the partial thermomagnetic curves, which suggest maximum temperatures below 500°C. Although the ash (WL facies) and possibly also the substrate’s surface reached high temperatures (> 600°C), lower temperatures inside the black layer and carbonate crust can be explained by the insulating effect of the ashes limiting the penetration of heat in depth [70,91], among other factors. Furthermore, there is significant lateral and vertical temperature variability in combustion structures such as the ones studied here, supporting co-existence of different magnetization mechanisms (full TRM, TCRM and pTRM) in different parts of the same combustion structure.

### 4.3. Preservation of the magnetic signature and taphonomic conditions

Certain behaviours of the thermomagnetic curves can be interpreted in terms of taphonomic processes. Ashes are variable showing both high thermomagnetic reversibility and irreversibility in some cases. A stable and highly reversible thermomagnetic behaviour is expected given their associated high temperatures (> 600°C). Irreversibility can be explained as a result of certain taphonomic processes such as trampling, animal bioturbation or other processes that enhance reworking of ashes with underlying lightly burnt or overlying unburnt substrates. During excavation of the open-air combustion structures, it was difficult to identify ash. Also, grayish-brown aggregates (probably a mixture of sediment and ashes) were observed on top of the combustion structures, presumably as a result of earthworm activity. Plant growth in the open-air combustion structures was also observed (S4 Fig). Regarding cave-entrance combustion structures, the ashes were reasonably well-preserved but not physically *in situ* given their high volatility. Assessing the preservation potential of ash is important for archaeomagnetic analysis because it contains the highest concentrations of ferromagnetic minerals. Unfortunately, our data corroborate that in the contexts tested, ash layers are not suitable for archaeomagnetic directional analyses since their *in situ* nature cannot be guaranteed.

Exceptionally high values of magnetization observed in IRM results of NFT-9 BL are also interpreted as a result of taphonomic conditions. Surprisingly, these values are even higher than those from ashes of other combustion structures. Keeping in mind that this combustion structure was intensively trampled for 15 days just after the firing, it is suggested that trampling incorporated ash into the subjacent BL consequently enhancing its magnetic mineral concentration. Mallol *et al*. [32] showed micromorphological evidence of ash inclusions in the BL of an experimental combustion structure trampled for 21 days.

Regarding to the directional dataset, it is expected that taphonomic processs as those observed (mainly bioturbation) affect the magnetic record due to mechanical sedimentary disturbance. However, directional results show considerable high quality. The angular distance (β) between each ChRM mean direction and the expected direction of the EMF at the experiment location oscillates between 0.6° and 7.3°. In combustion structures NFT-20-33, NFT-21 and NFT-22, the expected direction is within α95. This is not the case for NFT-9 and NFT-18. However, in all cases the difference between β and α95 (β-α95) is < 2°. This value can be considered to fall within the estimation error for the EMF at the studied location (obtained from the IGRF-12 model [83]). Subsequently, the errors can be considered stochastic and no statistically significant systematic error was found. In all cases, deviation can be explained as caused by stochastic dispersion related to subsampling, measurement errors, mechanical disturbance related to taphonomic processes, etc.

Precision parameter *k* is also informative of the quality of the directions, since it reflects the dispersion of the sample collection. When *k* values are higher, the population is better grouped. In our dataset, values are variable, although generally good. In the cases of NFT-18 and NFT-20-33, *k* values are exceptionally high (Table 2).

Directional analyses were exclusively developed on specimens of BL and burnt carbonate crust substrates. The BLs were relatively compacted before heating, and the burnt carbonate crust was lithified. Thus, they are less susceptible to reworking than ashes. Such higher degree of compaction and consolidation might explain the good state preservation observed. Consequently, selection of the facies for archaeomagnetic analyses is a key methodological aspect. Combustion substrate facies seem to be better candidates than ashes.

In spite of the general good quality of the directions, there is room for some remarks. Below, we discuss each combustion structure individually in order to interpret our archaeomagnetic results considering their specific taphonomic conditions.

The inclination obtained in open-air combustion structure NFT-9 is somewhat higher than the expected (Fig 10A). Trampling should have caused a shallowing effect with lower inclination values, but this is not the case. The precision parameter (*k* = 79.5) is statistically rather acceptable and the discrepancy between the obtained and expected direction might be explained by the error associated to sampling, sub-sampling and/or measuring. The most striking result is that a reliable archaeomagnetic direction was obtained from the BL facies even though this combustion structure was intensively trampled. Thus, the compact, more consolidated original state of the BL facies compared to the loose WL facies (ash) is a factor to consider in assessing the preservation potential of different facies within a combustion structure.

Open-air combustion structure NFT-18 shows a very low deviation of the archaeomagnetic direction (β = 3.6°) and the highest precision parameter (*k* = 565.3; Table 2 and Fig 10B). Such a small deviation is interpreted as a result of sampling/sub-sampling error but the quality of the palaeomagnetic direction and the statistical parameters are unquestionable.

Open-air combustion structure NFT-22 displays the poorest statistical results (*k* = 31.8) and maximum deviation from the expected direction (β = 6.1°) although the expected direction is within the α95 (β <α95; Table 2 and Fig 10C). As the sampling and subsampling processes here were the same as for the other combustion structures, the cause of this higher dispersion might be linked to taphonomic processes. The interpretation of its NRM demagnetization diagrams was complex. Its Q_n_ ratio values are the lowest among the open air burnt samples (Fig 2C and 2D), suggesting that the magnetization record is not very efficient. Moreover, the maximum T_UB_ of the ChRM direction (pTRMs) tends to be relatively low (*ca*. 150–300°C). Interestingly, during its excavation the identification of the burnt facies was not as clear as in the other combustion structures. With the exception of NFT-9 which was intensively trampled, all open-air combustion structures underwent similar taphonomic processes. We therefore interpret the poor directional results of NFT-22 due to a lower thermal impact in the sampled areas. Notwithstanding, a poor preservation cannot be totally excluded

The archaeomagnetic results of cave entrance combustion structure NFT-21 are conditioned by breakage of the most heated part of one of the blocks, resulting in weak and erratic NRM diagrams (e.g.: Fig 11C). This fire was extinguished with sediment, which would have presumably affected the ashes but not the underlying (lithified) burnt carbonate substrate. Nonetheless, despite the relatively high uncertainty due to the low number of available specimens (α95 = 7.1°; Table 2), α95 contains the expected direction (β < α95; Fig 10D).

Finally, cave entrance combustion structure NFT-20-33 gave us the opportunity to explore archaeomagnetic properties in relighting events, as it is a fire made directly on top of a preceding one carried out three years before. The high temperatures achieved in both fires and the lithified nature of the carbonate substrate facilitated a suitable record of the Earth´s magnetic field (β < 1°, Fig 10E) and the *in situ* preservation of the burnt surface. The statistical results are very good (*k* = 288.1). Some authors have shown that reheated structures are likely to yield better archaeomagnetic results than a single heating even at high temperature [92]. Reheating was not detected in the magnetic signal for two main reasons: First, if the last heating reaches high enough temperatures, the previous magnetization will be reset [93]. Second, the local Earth’s magnetic field direction in 2010 and 2013 was so similar that is statistically impossible to distinguish such variation in our results.

Our results reinforce the idea that archaeomagnetism and rock magnetism are two promising tools for the study of prehistoric fire. Although poor results should be expected considering the observed taphonomic conditions, the preservation of the magnetic signature is very good. The reliability of the EMF’s directional record on the studied combustion structures supports the application of archaeomagnetic analyses in Palaeolithic contexts, which can be helpful to assess geochronological questions. At the same time, analysis of magnetic signature provides information on estimation of maximum temperatures and thus contribute to the thermal characterization of Paleolithic fires by combining this information with the data obtained from other disciplines. Furthermore, bearing in mind the general good directional results despite the taphonomic processes observed, detection of very deviated and scattered mean archaeomagenetic directions in Palaeolithic combustion structures may be linked to mechanic post-depositional processes of higher impact than those detected here. Such directional behaviour might also be related to low heat impact if pTRM with low unblocking temperatures are detected and the macroscopic evidence of heating is ambiguous.

## 5. Conclusions

The following conclusions can be obtained from our study of five experimental combustion structures:

- Combustion structures must be analysed in terms of type of substrate and facies to fully understand the final magnetic outcome.
- Ashes show the highest concentration of ferromagnetic minerals followed by black layers (BL) and burnt carbonate crusts. The main magnetic carrier was pseudo-single domain (PSD) slightly substituted magnetite.
- In spite of the high concentration of ferromagnetic minerals in ashes and their good preservation in the cave entrance combustion structures, ashes are not the most suitable facies for archaeomagnetic directional analyses due to their propensity towards physical reworking, in contrast to more consolidated combustion substrate facies
- A decreasing pattern of NRM intensities and magnetic susceptibility was observed in black sedimentary layers and burnt carbonate crusts as a function of thermal impact.
- Most black layer and carbonate crust samples have systematically recorded pTRMs as a result of mild heating undergone by these facies in the field experiment. However, samples carrying a full TRM or TCRM were also detected. These different behaviours are consistent with the lateral and vertical variability of temperatures in this kind of structures, since the mechanism of magnetization is mainly controlled by the temperatures reached and the mineralogical or granulometric changes involved. Regardless of whether the mechanism is thermal or thermochemical, in both cases the directional record takes place in presence of the same field and thus it is reliable.
- When pTRMs are identified, the combination of NRM thermal demagnetization and partial thermomagnetic curves is a useful approach to estimate maximum last heating temperatures.
- High quality archaeomagnetic directions were obtained from BLs and burnt carbonate crusts of the open-air and cave entrance combustion structures respectively. The moderately compacted nature of the BLs and the lithified character of the burnt carbonate crusts favoured their *in situ* preservation.
- Generally, systematic deviations in directions were not larger than the uncertainty derived from stochastic dispersion.
- The thermally altered substrate of the combustion structure (excluding ashes) trampled for two weeks showed very good directional results and no significant distortion caused by that action.
- There is a correlation between well-preserved combustion structures and good archaeomagnetic results. The poorest results (multicomponent and erratic directional behaviours, low NRM intensities and *k* values (*k* < 40)), were obtained from combustion structures in which the thermal impact was unclear during their excavation or in which we know that the most heated part was lost. These results can be useful indicators of taphonomic alterations and/or low heat impact in similar archaeological combustion structures.
- The reheated combustion structure showed a good directional record. However, relightings cannot be distinguished if the last heating reached high temperatures to reset the previous magnetization and/or if the directions of the local Earth’s magnetic field at the time of the heating(s)/cooling(s) are so similar as to be statistically differentiated.

## Supporting information

S1 Text Detailed description of the studied combustion structures. (DOC)

S1 Fig Pictures of the studied structures after the combustion. (A-B) NFT-9 (A: conditions immediately after the end of the combustion; B: conditions after the addition of burnt bone from other fire and after trampling), (C) NFT-18, (D) NFT-20, (E) NFT-33, (F) NFT-21, (G) NFT-22. In the cases of NFT-21 and NFT-22, as there are no pictures of that moment, the images included are the available photographs closest to the end of the combustion and post-combustion actions, taken around three months later.
(TIF)

S2 Fig Examples of specimens from the hearth NFT-20-33, showing the different facies observed. (TIF)

S3 Fig Detail of a block from NFT-21, showing the presence of oncolites. A colour degradation pattern from the top (most heated, upper part of the image) to the bottom (less heated, lower part of the image) can be observed
(TIF)

S4 Fig Vegetation grown over the area where open-air combustion structures were carried out. (TIF)

S5 Fig Location of the oriented hand-blocks within each hearth. (A) NFT-9, (B) NFT-18, (C) NFT20-33, (D) NFT-21, (E) NFT-22. The orange/white transversal cord indicates approximately the middle of the hearth and the stars mark the location of the hand blocks for archaeomagnetism.
(TIF)
