# Supplementary material for: An experimental approach to the preservation potential of magnetic signatures in anthropogenic fires
Source: PLoS One. 2019 Aug 29;14(8):e0221592. doi: 10.1371/journal.pone.0221592 (PMC6715203; doi:10.1371/journal.pone.0221592)
Supplement: S1 Text — (DOC) [file pone.0221592.s001.doc]

**S1 Text. Detailed description of the studied combustion structures**

1. **NFT-9**

Open-air combustion structure located in the area close to the archaeological site (Fig 1 and S1 Fig). The substrate was a dry unvegetated soil, formed by loose silt on the surface with common angular limestons fragments from 1 to 3 cm. No thermocouple was available for this combustion structure.

The extinction of the fire was natural. The day after the combustion, a carbonized horse epiphysis from another combustion structure (NFT-6) was added. Moreover, NFT-9 was trampled for 16 days -starting the day after the combustion-. After that, the ash layer disappeared and the perimeter of the combustion structure began blurred. Combustion residues such ash charcoal were scattered around the combustion structure.

Then, NFT-9 was exposed during 5 years and, as well as the other open air combustion structures, it suffered the weather effects and plants rapidly grew over it (S4 Fig**)**. During the excavation, *Celtis* leaves and seeds, around 1 cm of dry grass, charcoal fragments (1-5 cm) and greyish-brown soil aggregates (2-5 cm) were detected above the open-air area combustion structures NFT-9, NFT-18 and NFT-22.

1. **NFT-18**

NFT-18 was located in the same area of NFT-9 and NFT-22 combustion structures (described below) so the substrate is the same (Fig 1 and S1 Fig). The extinction of the fire was natural and no anthropic action took place after it. NFT-18 was exposed during 5 years. There are data from two thermocouples that reached maximum temperatures of 663°C next to the flames and 622°C in the surface. Some roots were observed inside the sampled blocks.

1. **NFT-20-33**

NFT-20-33 is actually a set of two combustion structures performed inside of a cave: NFT20 and NFT-33 (Fig 1 and S1 Fig). NFT-20 was done in 2010 and three years later, NFT-33 was lit just exactly on top of NFT-20. The goal of this was to assess the possibility of distinguishing the magnetic signals of different combustion structures carried out in the same place with three years of difference. The original substrate was cemented carbonate-rich silt (carbonate crust, about 1-2 mm of thickness), with an underlying detrital layer (variable thickness) (S3 Fig). Sometimes, the upper part of the detrital layer seems to be a little bit cemented (S3 Fig). In one of the blocks, another carbonate layer is observed below the detrital layer (S3 Fig).

NFT-20 extinction was natural. No anthropic action was performed after the combustion, until the lit of NFT-33. The extinction of NFT-33 was natural and no post-combustion action was carried out.

Three thermocouples were available from NFT-20 and other three from NFT-33. One thermocouple from NFT-20 was located in the embers-flame interface and reached 690°C. The other two were located in the peripheral area of the surface and reached 770 and 733°C. The three thermocouples from NFT-33 recorded maximum temperatures of 101°C, 592°C and 864°C respectively.

1. **NFT-21**

NFT-21 was located inside of the cave (Fig 1 and S1 Fig). The substrate was cemented carbonate-rich silt (carbonate crust) and millimetric to centimetric-size oncolites appeared below (S3 Fig). This fire was extinguished with sediment and no post-combustion action was carried out. After the extinction of the fire, NFT-21 was exposed during 5 years.

Six thermocouples were available here. One was located in the embers-flame interface, and reached a maximum temperature of 763°C. The others were distributed in different parts of the combustion structure surface and their maximum temperatures oscillated among 53°C and 706°C.

1. **NFT-22**

NFT-22 was located next to NFT-9 and NFT-18 (Fig 1 and S1 Fig) in the same substrate described above. No post-combustion action was developed and NFT-22 was exposed during 5 years. Six thermocouples recorded temperatures. One attached to the embers reached a maximum temperature of 846°C. The other five thermocouples located on the surface reached maximum temperatures ranging from 218°C to 868°C.

After the exposure time, the ash layer was affected by the growth of grass above it. Furthermore, during the excavation of NFT-22, the signs of combustion (e.g.: thermoaltered facies) were not evident. Maybe the thermal impact was insufficient in some parts or the conservation of the burnt facies was limited.
