# Supplementary figures and images for: An experimental approach to the preservation potential of magnetic signatures in anthropogenic fires
Source: PLoS One. 2019 Aug 29;14(8):e0221592. doi: 10.1371/journal.pone.0221592 (PMC6715203; doi:10.1371/journal.pone.0221592)

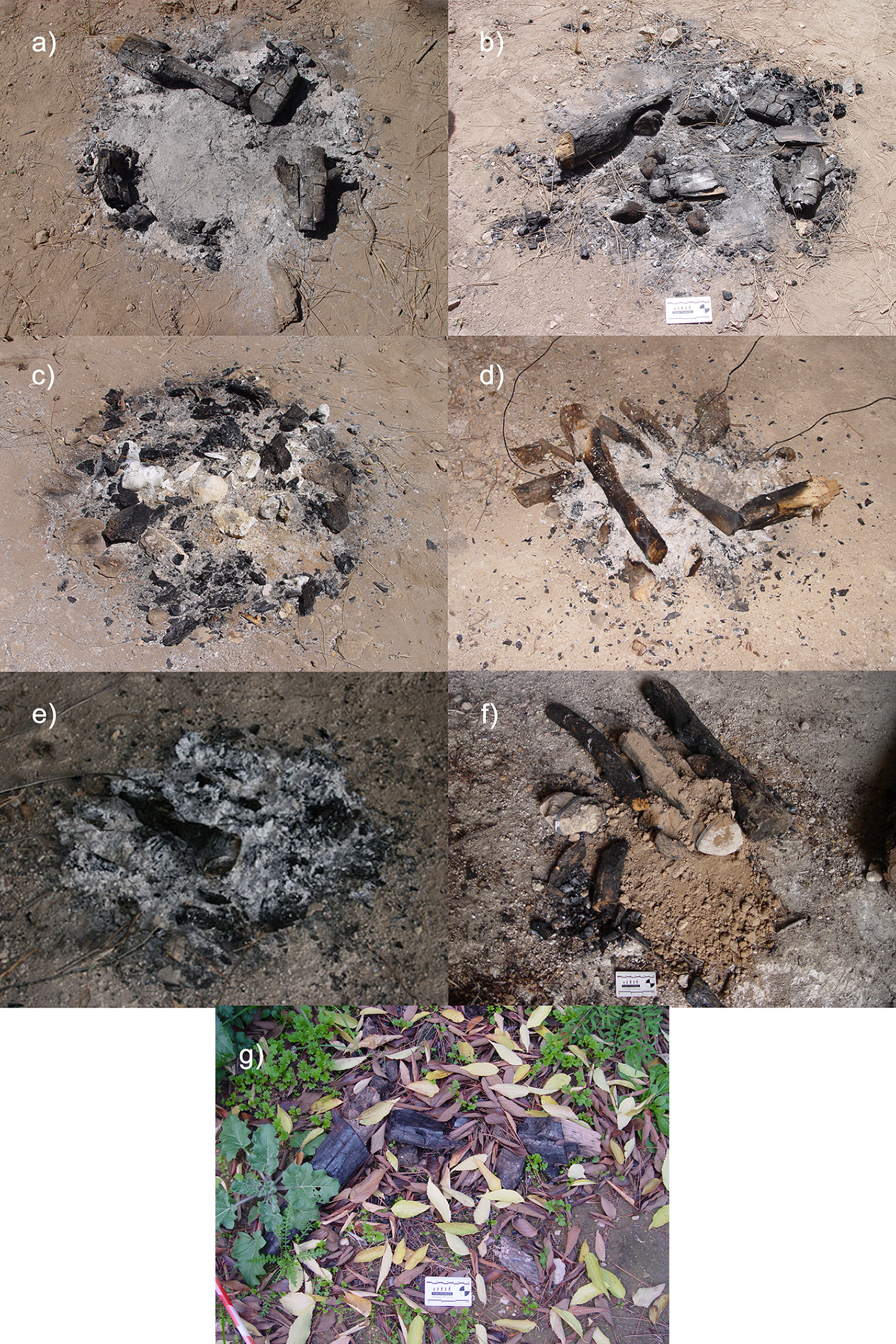

Supplement: S1 Fig — (A-B) NFT-9 (A: conditions immediately after the end of the combustion; B: conditions after the addition of burnt bone from other fire and after trampling), (C) NFT-18, (D) NFT-20, (E) NFT-33, (F) NFT-21, (G) NFT-22. In the cases of NFT-21 and NFT-22, as there are no pictures of that moment, the images included are the available photographs closest to the end of the combustion and post-combustion actions, taken around three months later. (TIF) [file pone.0221592.s002.tif]

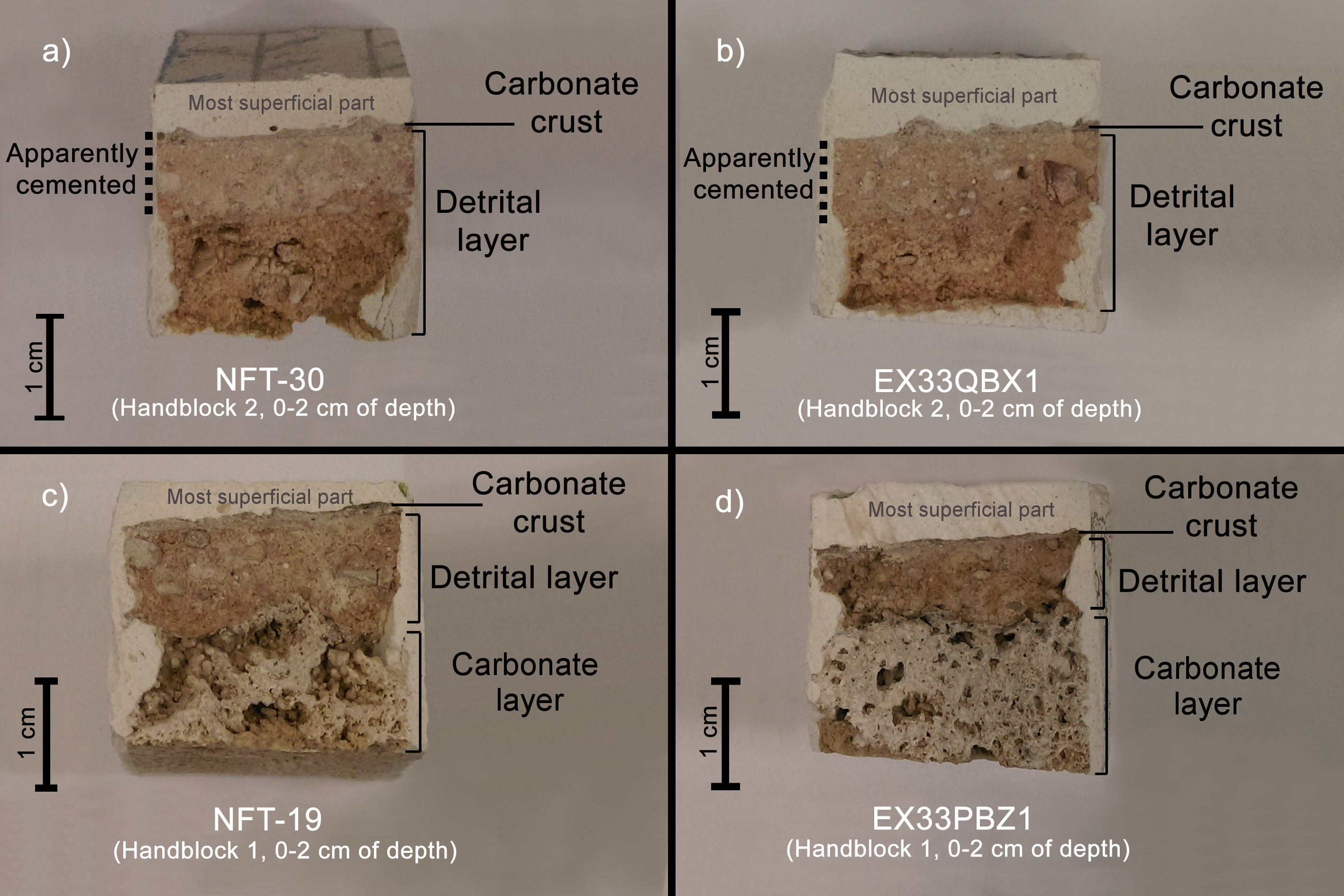

Supplement: S2 Fig — (TIF) [file pone.0221592.s003.tif]

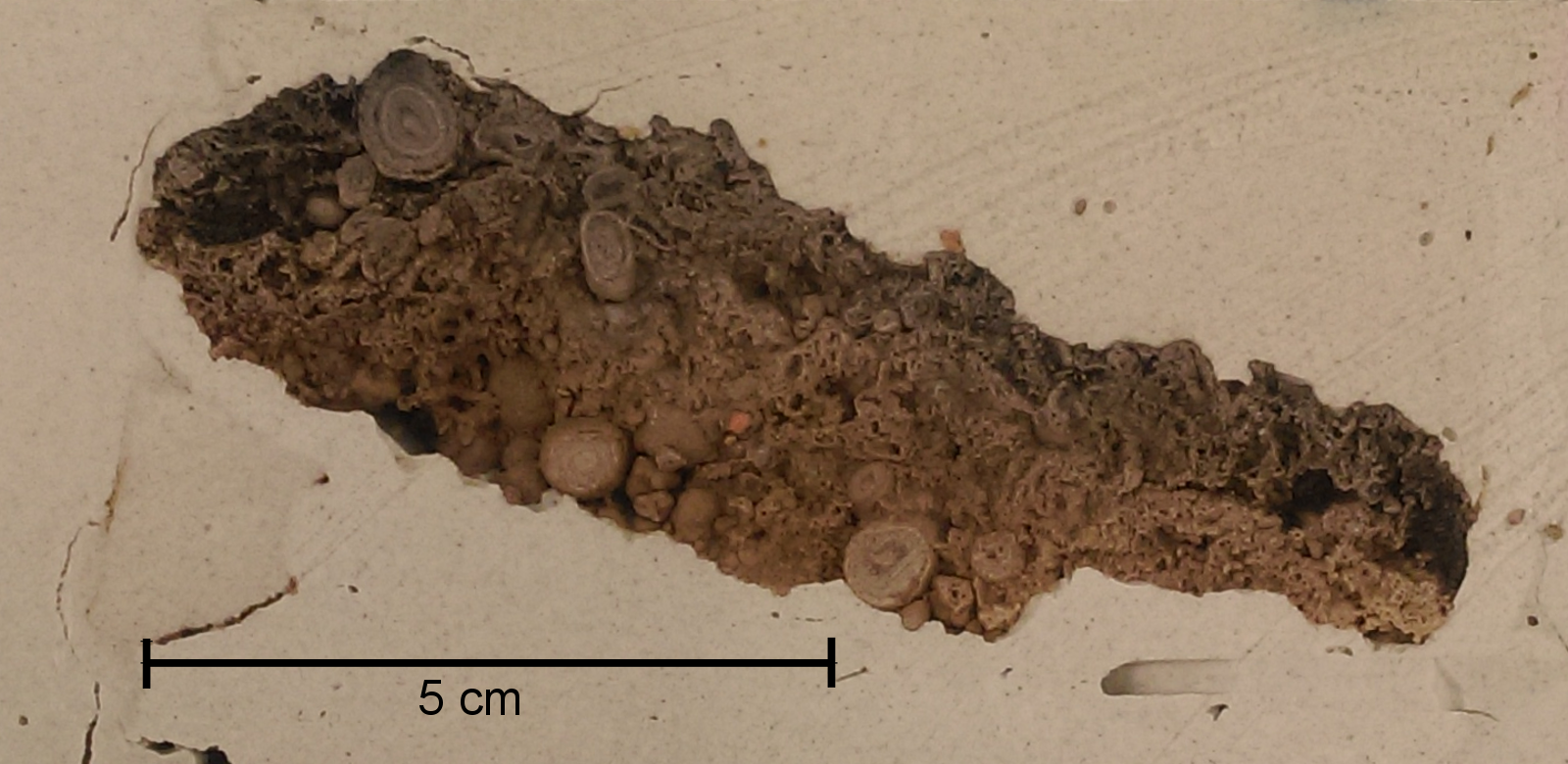

Supplement: S3 Fig — A colour degradation pattern from the top (most heated, upper part of the image) to the bottom (less heated, lower part of the image) can be observed (TIF) [file pone.0221592.s004.tif]

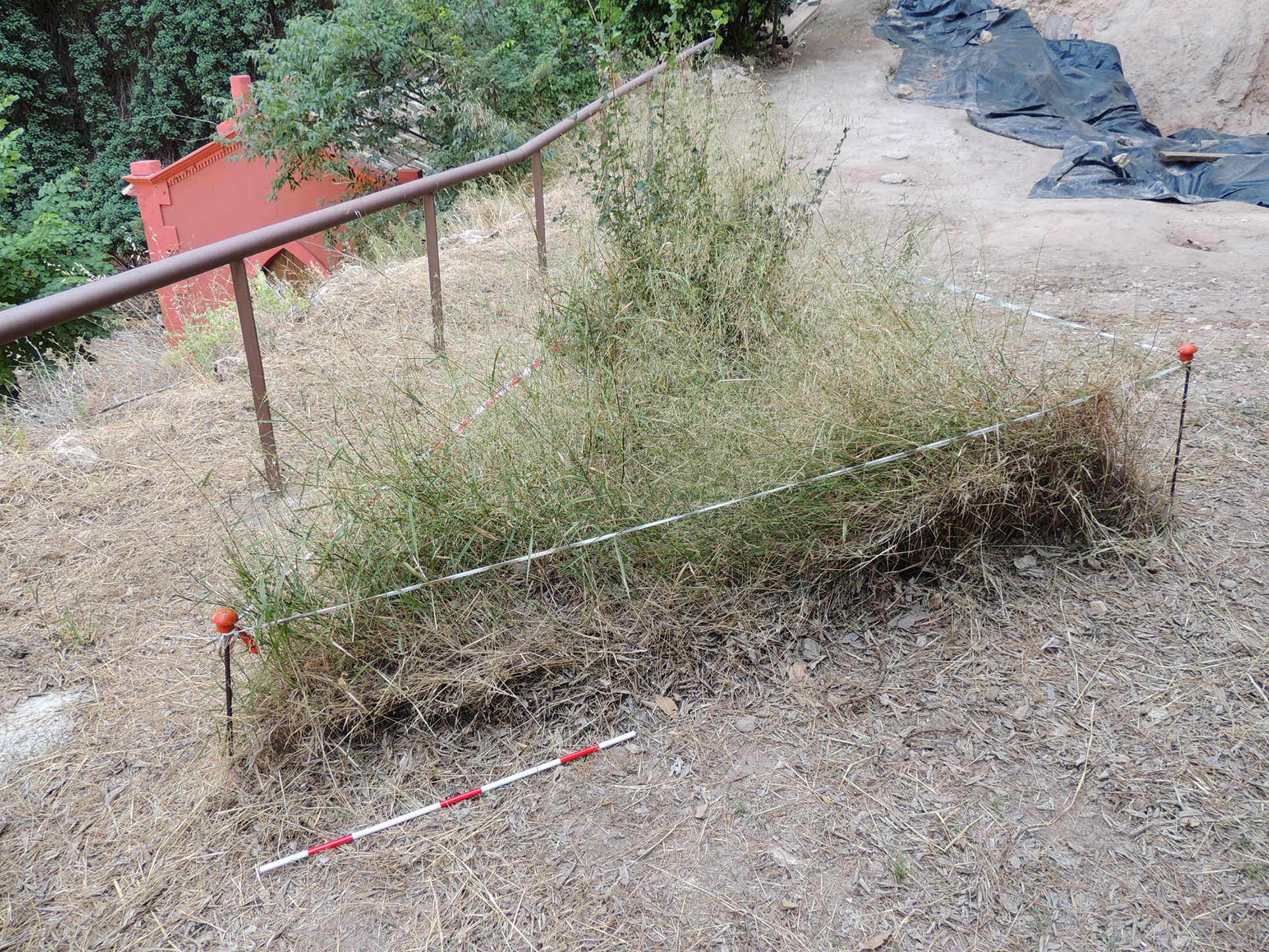

Supplement: S4 Fig — (TIF) [file pone.0221592.s005.tif]

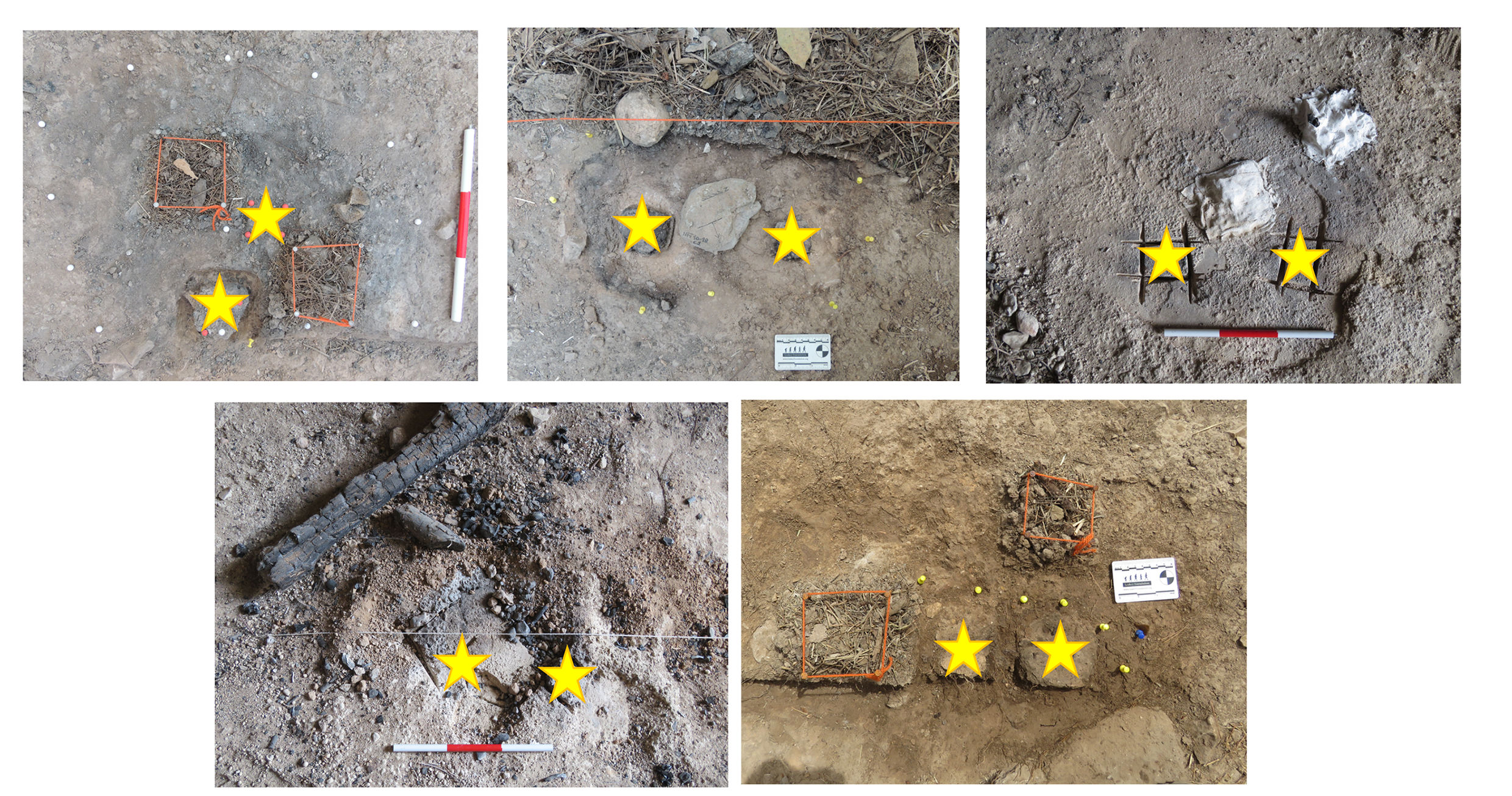

Supplement: S5 Fig — (A) NFT-9, (B) NFT-18, (C) NFT20-33, (D) NFT-21, (E) NFT-22. The orange/white transversal cord indicates approximately the middle of the hearth and the stars mark the location of the hand blocks for archaeomagnetism. (TIF) [file pone.0221592.s006.tif]
